# Metformin Reverses the Enhanced Myocardial SR/ER–Mitochondria Interaction and Impaired Complex I-Driven Respiration in Dystrophin-Deficient Mice

**DOI:** 10.3389/fcell.2020.609493

**Published:** 2021-01-25

**Authors:** Claire Angebault, Mathieu Panel, Mathilde Lacôte, Jennifer Rieusset, Alain Lacampagne, Jérémy Fauconnier

**Affiliations:** ^1^PhyMedExp, Université de Montpellier, INSERM, CNRS, Montpellier, France; ^2^CarMeN Laboratory, Inserm, INRA, INSA Lyon, Université Claude Bernard Lyon 1–Univ Lyon, Lyon, France

**Keywords:** Duchenne muscular dystrophy cardiomyopathy, mitochondria-associated ER membrane, mitochondrial calcium uniporter, MICU1, calcium

## Abstract

Besides skeletal muscle dysfunction, Duchenne muscular dystrophy (DMD) exhibits a progressive cardiomyopathy characterized by an impaired calcium (Ca^2+^) homeostasis and a mitochondrial dysfunction. Here we aimed to determine whether sarco-endoplasmic reticulum (SR/ER)–mitochondria interactions and mitochondrial function were impaired in dystrophic heart at the early stage of the pathology. For this purpose, ventricular cardiomyocytes and mitochondria were isolated from 3-month-old dystrophin-deficient mice (*mdx* mice). The number of contacts points between the SR/ER Ca^2+^ release channels (IP3R1) and the porine of the outer membrane of the mitochondria, VDAC1, measured using *in situ* proximity ligation assay, was greater in *mdx* cardiomyocytes. Expression levels of IP3R1 as well as the mitochondrial Ca^2+^ uniporter (MCU) and its regulated subunit, MICU1, were also increased in *mdx* heart. MICU2 expression was however unchanged. Furthermore, the mitochondrial Ca^2+^ uptake kinetics and the mitochondrial Ca^2+^ content were significantly increased. Meanwhile, the Ca^2+^-dependent pyruvate dehydrogenase phosphorylation was reduced, and its activity significantly increased. In Ca^2+^-free conditions, pyruvate-driven complex I respiration was decreased whereas in the presence of Ca^2+^, complex I-mediated respiration was boosted. Further, impaired complex I-mediated respiration was independent of its intrinsic activity or expression, which remains unchanged but is accompanied by an increase in mitochondrial reactive oxygen species production. Finally, *mdx* mice were treated with the complex I modulator metformin for 1 month. Metformin normalized the SR/ER-mitochondria interaction, decreased MICU1 expression and mitochondrial Ca^2+^ content, and enhanced complex I-driven respiration. In summary, before any sign of dilated cardiomyopathy, the DMD heart displays an aberrant SR/ER-mitochondria coupling with an increase mitochondrial Ca^2+^ homeostasis and a complex I dysfunction. Such remodeling could be reversed by metformin providing a novel therapeutic perspective in DMD.

## Introduction

Duchenne muscular dystrophy (DMD) is the most common X-linked disorder (1/3,500 newborn male affected) caused by non-sense mutations in dystrophin gene and resulting in the absence of the large protein dystrophin (427 kDa) (Hoffman, 2020). Dystrophin links the cytoskeleton to a complex of proteins at the cell surface, which, in turn, interacts with the extracellular matrix. Dystrophin deficiency causes progressive muscle weakness and cardiac failure. Cardiac involvement is inevitable and progresses with age toward a dilated cardiomyopathy (DCM) with an increased frequency of ventricular arrhythmia and sudden cardiac death. Among DMD patients, the cardiac phenotype varies with age from no discernible cardiac remodeling or dysfunction to early onset of DCM with heart failure (Sasaki et al., 1998; Amedro et al., 2019; Segawa et al., 2020). Whereas more than 90% of DMD patients display echocardiographic features of left ventricular remodeling and contractile dysfunction by the age of 18 years 11/26/20 5:34:00 PM, in the murine model of DMD, the *mdx* mice, similar defects are evident at 42 weeks of age (Quinlan et al., 2004).

At the cellular level, altered calcium (Ca^2+^) homeostasis is one of the first pathophysiological features associated with dystrophin deficiency. Before any sign of DCM and cardiomyopathy, the calcium channels of the sarcoplasmic reticulum (SR), the type 2 ryanodine receptors (RyR2), are leaky due to posttranslational remodeling (Fauconnier et al., 2010). Associated with an increased Ca^2+^ influx due to sarcolemmal damages and overactivation of stretch-activated channels and/or L-type Ca^2+^ current, these defects increase the diastolic Ca^2+^ level and promote fatal cardiac arrhythmias (Williams and Allen, 2007a; Jung et al., 2008; Ullrich et al., 2009; Fauconnier et al., 2010; Prosser et al., 2011). In parallel, DMD is associated with a progressive deterioration of the mitochondrial ultrastructure and function (Kyrychenko et al., 2015). Mitochondrial defects were mainly characterized in skeletal muscle where a significant uncoupling of the oxidative phosphorylation, defects in complex I, and a reduction of ATP synthesis were observed (Sperl et al., 1997; Kuznetsov et al., 1998; Percival et al., 2013; Rybalka et al., 2014; Moore et al., 2020). In heart muscle, a metabolic shift from fatty acid oxidation to carbohydrate oxidation has also been observed prior to the onset of DCM and heart failure (Khairallah et al., 2007, 2008; Burelle et al., 2010). Furthermore, mitochondrial Ca^2+^ uptake as well as mitochondrial reactive oxygen species (ROS) production and oxidative damages are increased in the DMD *mdx* mouse model and contribute to the pathogenesis of heart failure in DMD (Williams and Allen, 2007b; Dubinin et al., 2020; Hughes et al., 2020). Importantly, the first signs of mitochondrial and bioenergetic deficiencies precede the decline in myocardial function. Therefore, targeting mitochondrial function and/or metabolism in DMD has become a therapeutic issue over the last decade. Among the therapeutic candidates, the antidiabetic drug metformin has recently been evaluated in DMD both in preclinical studies and in clinical trials (Ljubicic and Jasmin, 2015; Hafner et al., 2016, 2019; Mantuano et al., 2018; Vitiello et al., 2019). This N,N-dimethylbiguanide interferes with the activity of mitochondrial complex I and activates AMP-activated protein kinase (AMPK), a critical hub for metabolic-mediated signaling pathways (for a review, see Foretz et al., 2014). Although the variability of the severity profile and the different stages of the pathology will require larger-scale clinical trials, treatment with metformin in combination with nitric oxide precursors has shown encouraging positive effects, particularly on motor function (Hafner et al., 2019). In *mdx* mice, long-term treatment with metformin alone also improves diaphragm function and limits muscle damage and the development of fibrosis due to exercise (Mantuano et al., 2018). To date, the only evidence of a beneficial effect of metformin in the development of cardiomyopathy associated with DMD is a decrease in the mass index of the heart (Mantuano et al., 2018).

The tight connection between the sarco-endoplasmic reticulum (SR/ER) and the mitochondria, also called mitochondria-associated ER membrane (MAM), is essential for the maintenance of energy metabolism, Ca^2+^ homeostasis, and cell fate (Rossini and Filadi, 2020). The tethering is maintained by mitofusin-2 (MFN2) as well as by a macromolecular complex comprising the SR/ER Ca^2+^ channel IP3 receptor 1 (IP3R1) and the porine of the outer membrane of the mitochondria (OMM), VDAC1 (voltage-dependent anion channel 1). These two channels are linked by a chaperone, GRP75, forming a direct Ca^2+^ channeling between the SR/ER and the mitochondria (Lee and Min, 2018). To cross the inner mitochondrial membrane (IMM), Ca^2+^ passes through the mitochondrial Ca^2+^ uniporter (MCU) complex creating an IP3R1–GRP75–VDAC–MCU Ca^2+^ transfer axis (Lee and Min, 2018). MCU is a highly selective Ca^2+^ channel which comprises a membrane-spanning 40-kDa protein that forms a low-conductance Ca^2+^-selective pore. MCU exists as a large protein complex (~480 kDa) comprising MICU1 and MICU2 which give it its Ca^2+^ sensitivity (Tarasova et al., 2019). This SR/ER–mitochondria interaction and contact points have never been studied in DMD hearts. However, at the early stage of the pathology, we have recently demonstrated an impaired SR/ER–mitochondria interaction in *mdx* skeletal muscles associated with an alteration of Ca^2+^ homeostasis, and increase in the unfolding protein response (UPR or ER stress) and muscle weakness (Pauly et al., 2017).

The aim of this study was thus to establish whether, in *mdx* mice that show no signs of DCM, the interconnection between SR/ER and mitochondria is modified and whether it may interfere with myocardial mitochondrial function. Our results demonstrate that increased SR/ER–mitochondria interaction is associated with increased mitochondrial Ca^2+^ content and a disrupted mitochondrial function. One-month metformin treatment reversed these defects providing a novel therapeutic perspective in DMD.

## Methods

### Animal and Cell Isolation

All experiments were conformed to the institutional ethics committee for animal experiments and received the agreement from the national *Ministère de l'enseignement supérieur et de la recherche* (N° #16473-2018082016141320). Male *mdx* or WT (wild-type, C57bl/10ScSn) mice (CNRS, IGMM, France) were used at 3 months of age. According to the animal-to-human translation factor for drug treatments and as previously shown, a daily dose of metformin of 200 mg/kg/day was given for 1 month in 50 ml of drinking water (Reagan-Shaw et al., 2008; Mantuano et al., 2018). Mice receiving metformin treatment were housed in a single cage to ensure homogeneity between groups. Left ventricular myocytes were enzymatically dissociated as previously described (Fauconnier et al., 2005). In brief, after removal, the heart was retrogradely perfused at 37°C with a modified enzyme solution [0.1 mg.mL^−1^ of Liberase™ (Roche)] containing 113 mM NaCl, 4.7 mM KCl, 0.6 mM KH2PO4, 0.6 mM Na2HPO4, 1.2 mM MgSO4, 12 mM NaHCO3, 10 mM KHCO3, 10 mM Hepes, 30 mM taurine (pH 7.4). Isolated myocytes were then transferred to the same enzyme-free solution containing 1 mM CaCl_2_ prior experiments.

### Western Blot

Twenty to thirty micrograms of total or mitochondrial protein was separated on SDS-polyacrylamide gel and electro-blotted onto a nitrocellulose membrane (Bio-Rad). Membranes were saturated with blocking buffer for 1 h at room temperature and incubated overnight at 4°C with monoclonal mouse anti-VDAC (1:1000, Abcam), anti-OXPHOS (1:5000, Abcam), anti-IP3R1 (1:1000, Santa Cruz), anti-NDUFA13 (1:1000, Abcam), anti-GAPDH (1:10 000, Abcam) or with polyclonal rabbit anti-MCU (1:500, Abcam), anti-MICU1 (1:500, Thermo Fisher), anti-MICU2 (1:500, Sigma Aldrich), anti-PDH-E1α (1:1000, Abcam), anti-PDHE1α phosphor Ser 293 (1:1000, Abcam), anti-PDHE1α phosphor Ser 300 (1:1000, Millipore), anti-PDHE1α phosphor Ser 232 (1:1000, Calbiochem), anti-PDK4 (1:1000 Novus Biotech), anti-GRP75 (1:1000, Santa Cruz), anti-SIGMA1R (1:1000, Cell Signaling), anti-MFN2 (1:1000, Abcam), and anti-Hsp60 (1:1000, Abcam). Hsp60 and GAPDH were used as loading controls. All immunoblots were developed and quantified using the Odyssey infrared imaging system (LICOR Biosystems) and infrared-labeled secondary antibodies. Band intensities were quantified with ImageJ.

#### Quantitative Real-Time RT-PCR

Isolated cardiomyocyte RNA was extracted with the TRIzol Reagent (Life Technologies). The level of target mRNAs was measured by reverse transcription (Superscript II, Invitrogen, 1 μg total RNA) followed by quantitative real-time PCR using a RotorGene (Corbett Research). TATA-binding protein (TBP) mRNA was used as a housekeeping gene *Tbp* (forward 5′-TGGTGTGCACAGGAGCCAAG-3′, reverse 5′-TTCACATCACAGCTCCCCAC-3′). The primer sequences of ER stress-related genes are *Hspa5* forward 5′-CCACCTCCAATATCAACTTG-3′, *Hspa5* reverse 5′-ACGATCAGGGCAACCGCATCA-3′; *Ddit3* forward 5′-CTGGAAGCCTGGTATGAGGA-3′, *Ddit3* reverse 5′-CTCTGACTGGAATCTGGAGA-3′; total *Xbp1* forward 5′-GTGCAGGCCCAGTTGTCACC-3′, *Xbp1* reverse 5′-TCTGGGTAGACCTCTGGGAG-3′; U-*Xbp1* forward 5′-CAGACTATGTGCACCTCTGC-3′, U *Xbp1* reverse 5′-TCTGGGTAGACCTCTGGGAG-3′; *Atf3* forward 5′-CCAGGTCTCTGCCTCAGAAG-3′, *Atf3* reverse 5′-CATCTCCCAGGGGTCTGTTGT-3′; *Atf4* forward 5′-TCGATGCTCTGTTTCGAATG-3′, *Atf4* reverse 5′*-*AGAATGTAAAGGGGGCAACC-3′; *Atf6* forward 5′-TCGAGGCTGGGTTCATAGAC-3′, *Atf6* reverse 5′-CTGTGTACTGGACAGCCATC-3′.

### Proximity Ligation Assay

The Proximity Ligation Assay (PLA) was performed as previously described (Paillard et al., 2013; Pauly et al., 2017). Cells were fixed with paraformaldehyde 4% and permeabilized at RT with 0.1% Triton-X100. After washing, they were incubated with blocking buffer for 30 min at 37°C. The blocking solution was removed before incubation of primary antibodies (anti-VDAC Abcam, ab1734, 1:200, and anti-IP3R1, Santa Cruz, sc-28614, 1:200, or anti-GRP75, Santa Cruz, sc-13967) overnight at 4°C. The cells were washed two times using PBS with 0.01% Tween. The two PLA probes 1:5 were prepared in antibody diluent 20 min before incubation for 1 h at 37°C. Next, cells were incubated with mix containing 5× ligation stock (diluted 1:5 in water) and 1× ligation solution (diluted 1:40) for 30 min at 37°C. Next, the cells were incubated with mix containing 5× amplification stock (diluted 1:5 in water) and polymerase (diluted 1:80) for 100 min at 37°C. Finally, the cells were washed with Dapi (diluted 1:500) in wash buffer B 1× and mounted using Dako fluorescent mounting medium (S3023) and analyzed using a fluorescence microscope (excitation: 594 nm, emission: 624 nm, magnification: 40×).

### Mitochondrial Isolation and Endogenous Mitochondrial Ca^2+^ Content

Mitochondrial isolation protocols were performed and adapted from Frezza et al. (2007). Briefly, left ventricles (LV) or isolated cardiomyocytes were homogenized with Teflon pestle in cold isolation buffer (pH 7.4, 225 mM Mannitol, 75 mM Sucrose, and 30 mM Tris with anti-protease 1×) and homogenized by 10 strokes with a motorized Potter Elvejhem (1500 rpm). Nuclei and cellular debris were pelleted at 800 g for 10 min at 4°C. Supernatants containing mitochondria were centrifuged twice at 9000 g for 10 min to pellet mitochondria. Mitochondria were resuspended in isolation buffer, and protein concentration was determined by a bicinchoninic assay kit. Isolated mitochondria pellets were diluted in HCl 0.6 N, homogenized, and sonicated. After incubation during 30 min in boiling water, mitochondria were centrifuged for 5 min at 10 000 g, and the supernatant was recovered. Ca^2+^ content in supernatant was determined spectrophotometrically (Tecan) using an o-cresolphthalein complexone assay according to the manufacturer's instructions (TECO Diagnostics). Results are expressed in mM of Ca^2+^ per μg of protein.

### Mitochondrial Respiration and Mitochondrial Ca^2+^ Uptake

Respiration was measured on digitonin-permeabilized cardiomyocytes (15 μg per million cells) in the respiratory buffer containing 10 mM KH_2_PO_4_, 3 mM MgCl_2_·6H_2_O, 0.5 mM EGTA, 20 mM taurine, 60 mM K-lactobionate, 20 mM HEPES, 110 mM sucrose, and 1 g/L BSA, pH 7.1. The respiratory rates of 10 000 cardiomyocytes were recorded at 37°C using a high-resolution Oxygraph respirometer (Oroboros, Innsbruck, Austria) after addition of different substrates. Briefly, the aerobic glycolysis pathway was stimulated by addition of pyruvate and malate. Pyruvate, the last substrate generated by glycolysis, and malate activate the Krebs cycle after their conversion in acetyl coenzyme A and oxaloacetic acid, respectively. Under these conditions, the complex I of the mitochondrial respiratory chain was activated by NADH produced. Complex II-driven respiration was obtained by addition of succinate and rotenone (to inhibit the complex I), and complex IV-driven respiration was permitted by addition of antimycin A (to inhibit the complex III), ascorbate and N,N,N′,N′-tetramethyl-p-phenylenediamine dihydrochloride (TMPD) to reduce cytochrome c. In some experiments, the free Ca^2+^ was set to 400 nM in the respiratory buffer using the Maxchelator program (https://somapp.ucdmc.ucdavis.edu/pharmacology/bers/maxchelator/). The respiratory coupling ratio (RCR) was measured in all conditions, and no differences between groups were observed (Supplementary Figure 1). Mitochondrial Ca^2+^ uptake was measured with Calcium Green (Invitrogen) with the O2k-Fluorescence LED2-Module on isolated mitochondria in the presence of thaspigargin (10 μM). The decay time constant of the calcium green fluorescence was measured after application of 25 μM extramitochondrial Ca^2+^ and reflects mitochondrial Ca^2+^ uptake.

### Enzymology

Activity of the pyruvate dehydrogenase (PDH) was obtained by using Pyruvate Dehydrogenase Enzyme Activity Microplate Assay (Abcam ab109902) and 100 μg of mitochondria isolated from heart homogenates according to the manufacturer's instruction. Mitochondrial enzymatic activities of complexes I and IV and citrate synthase were measured on mitochondria isolated from heart homogenates at 37°C using a SAFAS spectrophotometer and standard method (Angebault et al., 2018).

### ROS Measurement

Mitochondrial anion superoxide production was monitored using the fluorescent indicator MitoSOX Red (Invitrogen) and confocal microscopy as described previously (Fauconnier et al., 2007). Briefly, cardiomyocytes were loaded with MitoSOX Red (5 μM) for 30 min at room temperature, followed by washout. Confocal images were obtained after 5 min of 1-Hz stimulation in standard Tyrode solution and subsequently after addition of antimycin A (50 μM) to estimate the maximal complex I superoxide production capacity. MitoSOX Red fluorescence was measured in the same region of the cell at each time point. The signal from each cell was normalized to that immediately before pacing.

### Statistics

All data are presented as mean ± SEM. Normality of distribution, controlled by Agostino–Person omnibus normality test, not being respected; the Mann–Whitney test was used for all experiments. For multiple comparisons, the non-parametric Kruskal–Wallis test was applied. The significance level was set at α = 0.05.

## Results

### SR/ER–Mitochondria Interaction and Mitochondrial Ca^2+^ Uptake in *mdx* Cardiomyocytes

**Figure 1:**
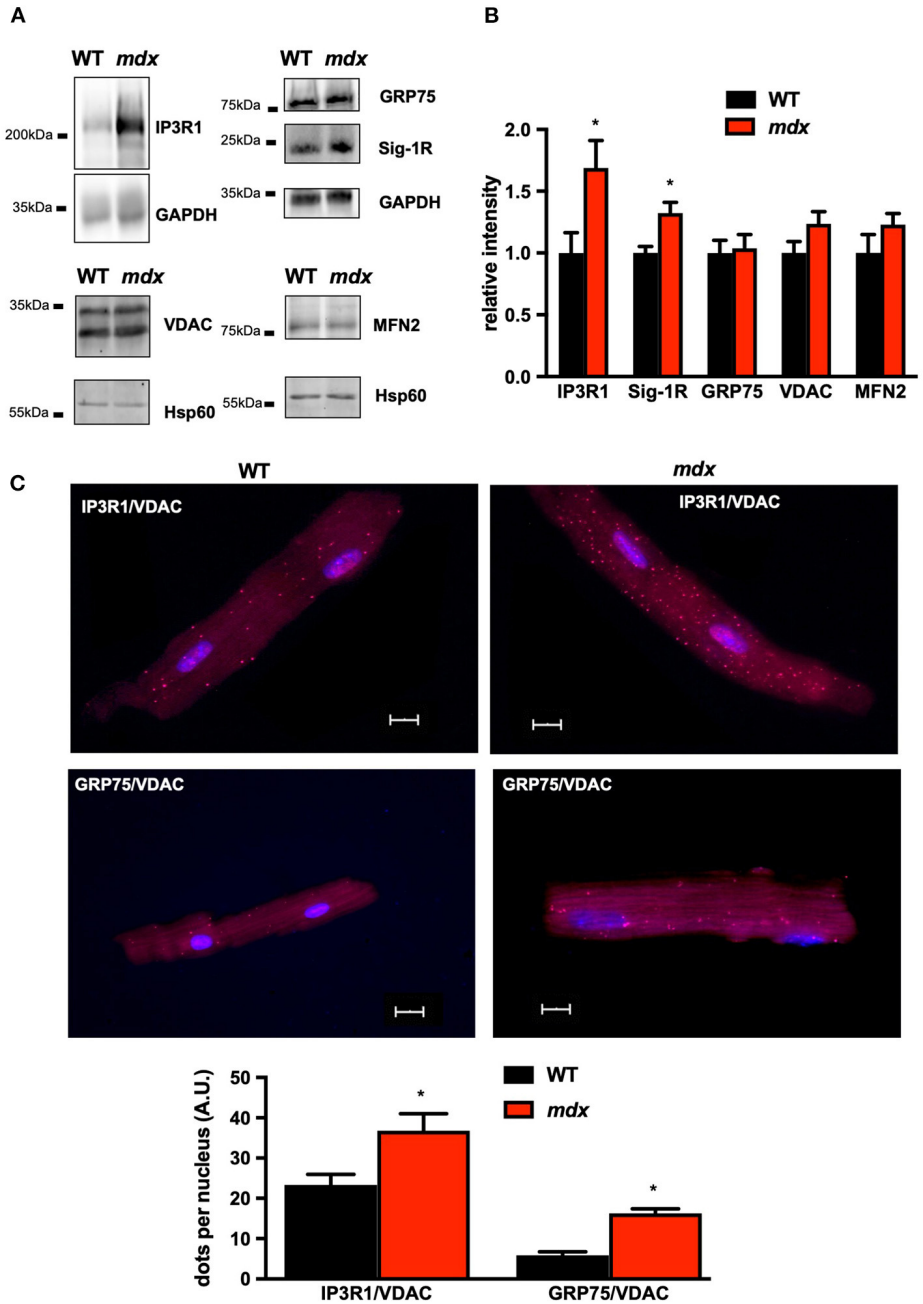
Increase in interconnection between SR/ER and mitochondria in *mdx* cardiomyocytes. All quantifications of proteins were normalized either to GAPDH when heart homogenates were used or to Hsp60 when Western blot were performed on isolated mitochondria and expressed relative to WT. **(A)** Representative immunoblots and quantification **(B)** of IP3R1, GRP75, Sig-1R, and VDAC. Quantification of IP3R1, GRP75, MFN2, and Sig-1R was carried out from *N* = 6 *mdx* and WT hearts and from *N* = 5 *mdx* and WT hearts for VDAC. Data are mean ± SEM. For IP3R1 *p = 0.0317 and for Sig-1R *p = 0.0043 *mdx* vs. WT. **(C)** Representative images and quantitative analysis of IP3R1-VDAC and GRP75-VDAC1 interaction measured by *in situ* PLA on isolated cardiomyoctytes from *mdx* (*N* = 3, *n* > 49) and WT (*N* = 3; *n* > 44). The numbers of dots were normalized to the number of nucleus per cells. Data are mean ± SEM. *p = 0.0057 *mdx* vs. WT for IP3R1-VDAC and *p* < 0.0001 *mdx* vs. WT for GRP75-VDAC1.

**Figure 2:**
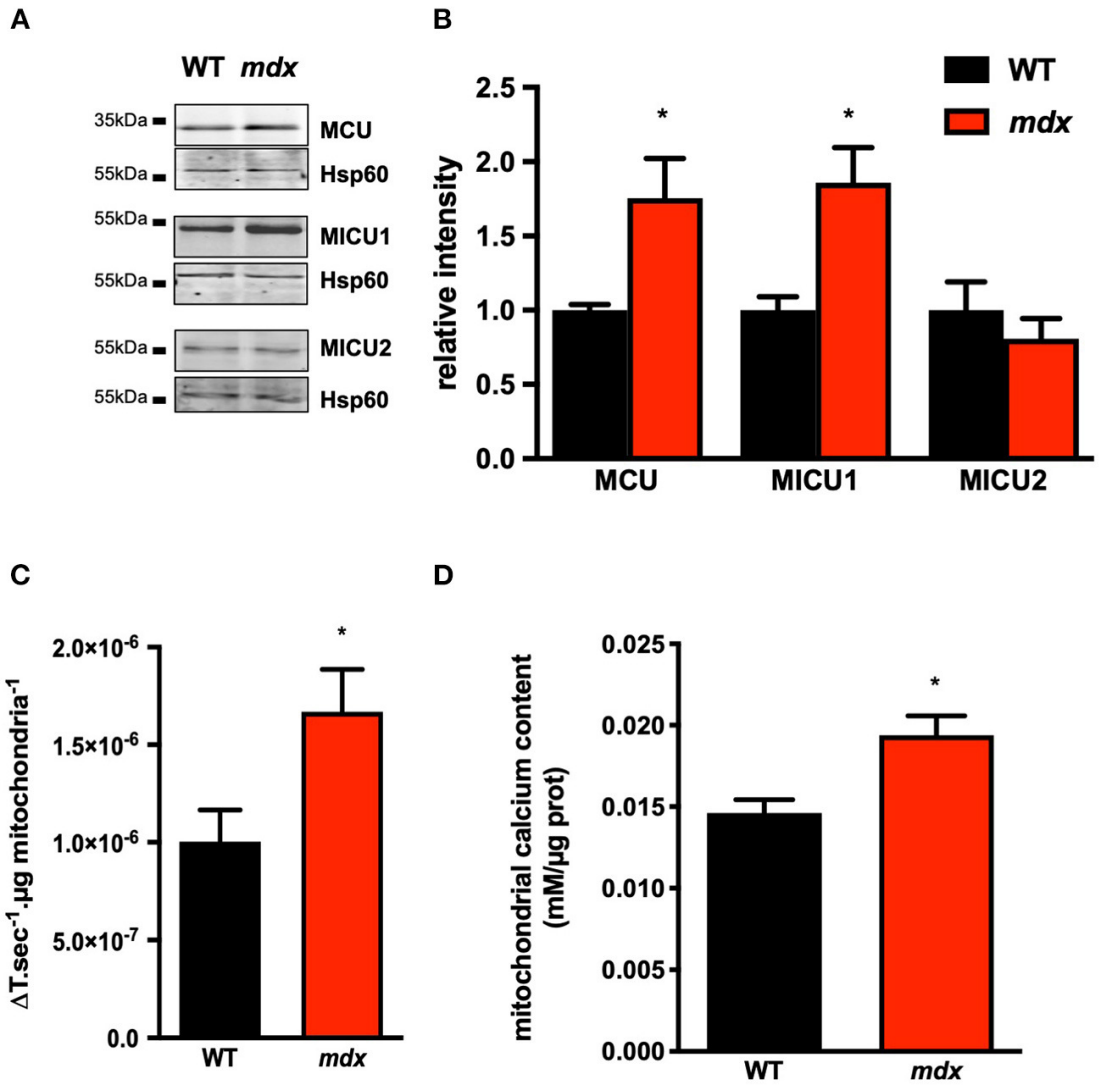
Increase MCU/MICU1 expression and mitochondrial Ca^2+^ in *mdx* cardiomyocytes. All Western blots were performed on isolated mitochondria and quantifications of proteins were normalized to Hsp60 and expressed relative to WT. **(A)** Representative immunoblots and quantification **(B)** of MCU, MICU1, and MICU2. Data are mean ± SEM, *p = 0.0043 and *p = 0.0022 for MCU and MICU1, respectively, *mdx* (*N* = 6) vs. WT (*N* = 6). **(C)** The mean rate of mitochondrial Ca^2+^ uptake measured as the decay time constant of calcium green fluorescence in presence of 25 μM of Ca^2+^. Data are mean ± SEM, *p = 0.0476 *mdx* (*N* = 6) vs. WT (*N* = 5). **(D)** Mean of the absolute mitochondrial Ca^2+^ content in *mdx* (*N* = 6) and WT (*N* = 6) hearts. Data are mean ± SEM, *p = 0.0152 *mdx* vs. WT.

We first determined whether SR/ER–mitochondria interactions are altered in mdx mouse cardiomyocytes as previously reported in skeletal muscle, namely, a decrease in SR/ER–mitochondria interaction (Pauly et al., 2017). For this purpose, we first measured the expression level of proteins involved in the SR/ER-mitochondria tethering. Although the MFN2 expression as well as VDAC1 and GRP75 levels were similar in both groups, IP3R1 and its regulatory subunits SIGMA-1R (Sig-1R) were significantly increased in *mdx* cardiomyocytes (Figures 1A,B). The expression of sarco/endoplasmic reticulum Ca^2+^-ATPase type 2a (SERCA2a) and its regulatory protein, phospholamban (PLB), and its phosphorylation were also unchanged, although the Ca^2+^ load of the SR was reduced (Supplementary Figure 2). We next evaluated the contact points between the two organelles using the *in situ* PLA assay. The IP3R1–VDAC1 interaction was significantly enhanced as well as the GRP75–VDAC1 connections, indicating that the physical SR/ER–mitochondria interconnection *via* IP3R1 and VDAC is enhanced in *mdx* cardiomyocytes (Figure 1C). However, the immunoprecipitation of IP3R1 did not show a difference in the VDAC/IP3R1 and GRP75/IP3R1 ratio meaning that the increase is at least in part related to the increase in the expression of IP3R1 (Supplementary Figure 3). In addition, except *Atf3* gene expression which increases, mRNA levels of ER stress markers were unchanged in *mdx* heart (Supplementary Figure 4). The increased IP3R1–GRP75–VDAC1 interaction is supposed to enhance the direct channeling for Ca^2+^ from the SR/ER to the mitochondria. This is reinforced by an increase in both MCU and MICU1 expression level as reported recently (Dubinin et al., 2020). Importantly, the MICU2 expression remained unchanged (Figures 2A,B). This structural remodeling was associated with a faster mitochondrial Ca^2+^ uptake indicating functional changing of the MCU complex (Figure 2C). Similarly, mitochondrial Ca^2+^ content was also significantly elevated compared to WT mitochondria (Figure 2D). Unlike skeletal muscle, these data indicate an increase in SR/ER–mitochondria interactions and mitochondrial Ca^2+^ uptake in the *mdx* heart.

### PDH Activity in *mdx* Cardiomyocytes

**Figure 3:**
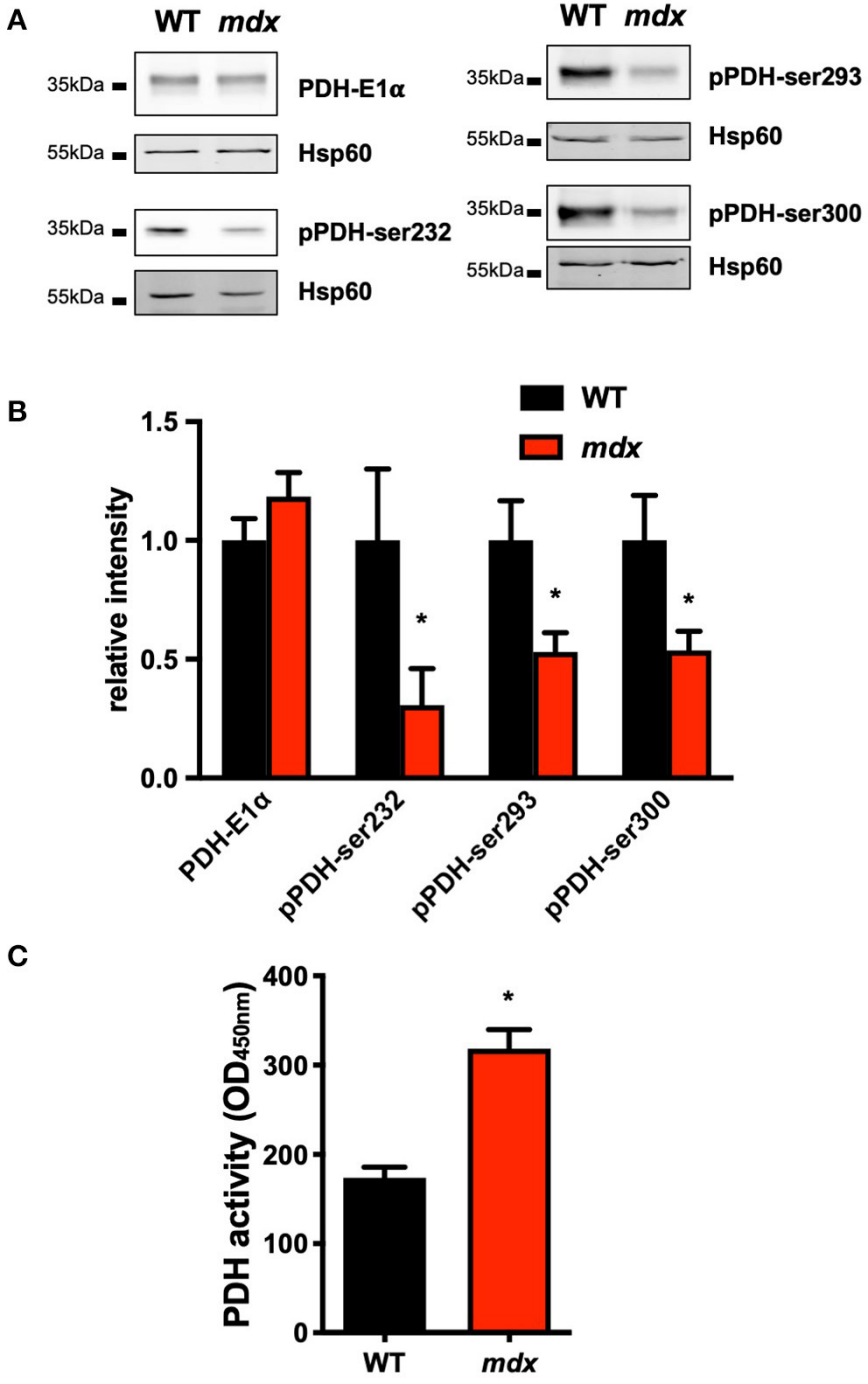
Increase PDH activity in *mdx* hearts. All Western blots were performed on isolated mitochondria and quantifications of proteins were normalized to Hsp60 and expressed relative to WT. **(A)** Representative immunoblots and **(B)** quantification of total pyruvate dehydrogenase subunit E1α (PDH-E1α), PDH phosphorylated on Serine 232 (*p = 0.0262), 293 (*p = 0.0499), and 300 (*p = 0.0281). Data are mean ± SEM, *mdx* (*N* = 8) vs. WT (*N* = 8). **(C)** Mean PDH activity measured on isolated mitochondria. Data are mean ± SEM, *p = 0.0043 *mdx* (*N* = 6) vs. WT (*N* = 6).

Once in the mitochondrial matrix, Ca^2+^ regulates several processes that interfere with the mitochondrial function and the metabolic flux. Among them, Ca^2+^ controls the activity of the PDH, which is the entry point for the glycolytic product pyruvate into the oxidative metabolism. The PDH phosphorylation by the PDH kinase (PDK) inhibits its activity whereas its dephosphorylation by the Ca^2+^-dependent PDH phosphatase (PDP) increases its activity. We thus measured the PDH phosphorylation in *mdx* LV mitochondria. The total PDH content and the PDK4 expression were comparable in both groups; however, the phosphorylation levels of the three phosphorylable serines (pPDH-ser232, pPDH-ser293, and pPDH-ser 300) were significantly reduced (Figures 3A,B; Supplementary Figure 5). Furthermore, the PDH activity was significantly increased in LV *mdx* mitochondria consistent with the decrease in PDH phosphorylation (Figures 3B,C).

### Ca^2+^ Regulates Oxidative Phosphorylation in *mdx* Cardiomyocytes

**Figure 4:**
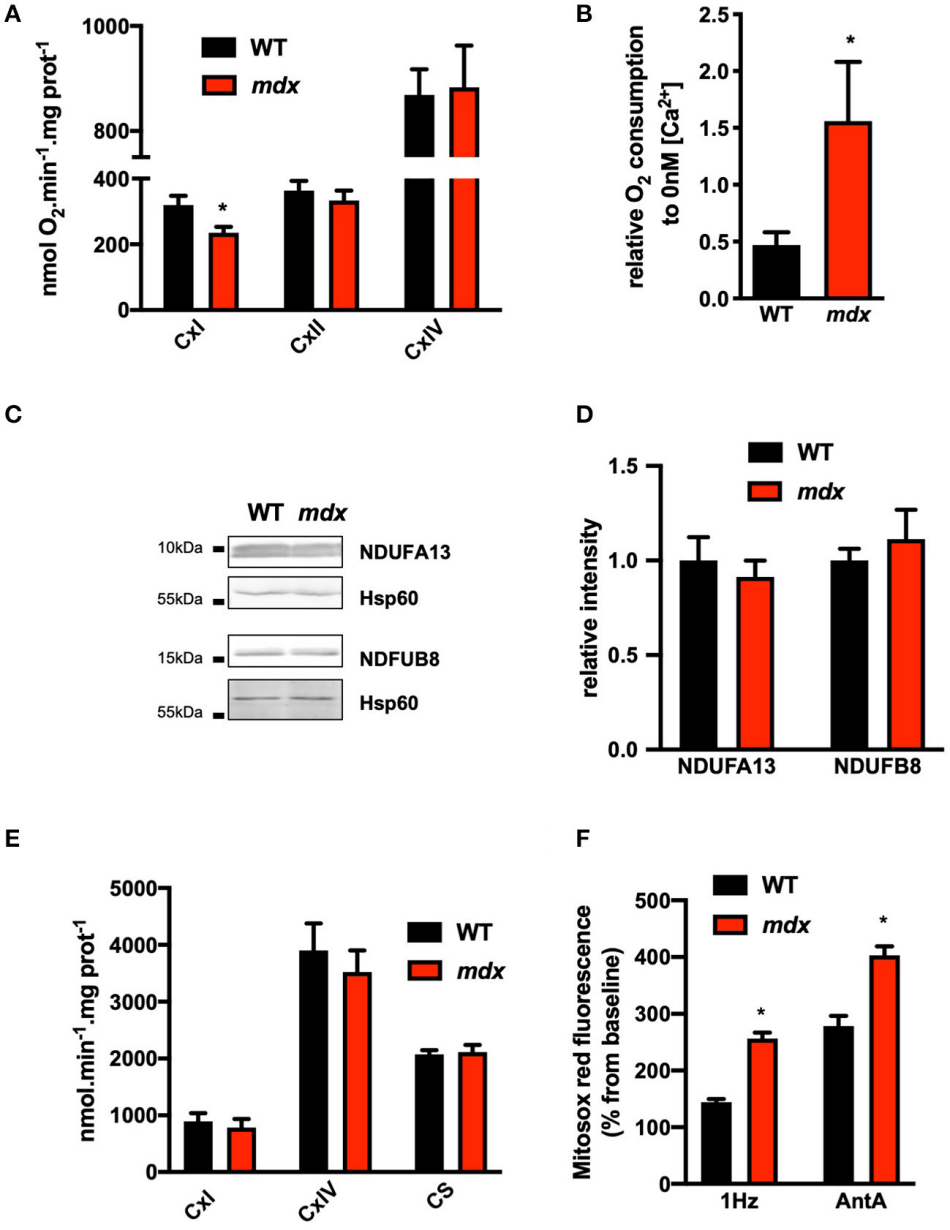
Impaired complex I-mediated mitochondrial respiration in *mdx* hearts. **(A)** Respiration rates under glycolysis protocols in permeabilized isolated ventricular cardiomyocytes from WT and *mdx* mice (*N* = 5–7). Complex I-dependent respiration State 3 (EIII) is determined in presence of malate and pyruvate (CxI). Complex II and IV-dependent respiration State 3 (EIII) is obtained by addition of succinate and rotenone (CxII) and ascorbate/TMPD (CxIV), respectively. Data are mean ± SEM, *p = 0.0379 *mdx* vs. WT. **(B)** Mean complex I-dependent respiration in the presence of 400 nM extramitochondrial Ca^2+^ relative to 0 nM Ca^2+^. Data are mean ± SEM, *p = 0.0260 *mdx* (*N* = 6) vs. WT (*N* = 6). **(C)** Representative immunoblots and **(D)** quantification of two subunits of complex I (NDUFA13: CxI 13 and NDUFB8: CxI 20). Isolated mitochondria from *N* = 6 hearts were tested in *mdx* and WT mice, and Hsp60 was used as loading control and expressed relative to WT. Data are mean ± SEM. **(E)** Enzymatic activities of complexes I and IV and citrate synthase measured on isolated mitochondria from Data are means ± SEM, *p* > 0.05 *mdx* (*N* = 5) vs. WT (*N* = 5). **(F)** Mitochondrial ROS production is evaluated under confocal microscopy with MitoSOX red and expressed as percentage of baseline after electric stimulation at 1 Hz (*p = 0.0071) or Antimycin A (*p = 0.0435) addition. Data are mean ± SEM, *mdx* (*N* = 10) vs. WT (*N* = 9).

Increased PDH activity may impact carbohydrate metabolic flux and pyruvate-mediated oxidative phosphorylation. We next measured maximal mitochondrial respiration rates in a Ca^2+^-free conditions using pyruvate/malate as carbohydrate substrates to feed the complex I. In the presence of ADP, the complex I-mediated oxygen consumption was significantly reduced whereas complex II- and complex IV-related respiration and the RCR were comparable in both groups (Figure 4A; Supplementary Figure 1). These results indicate that only the complex I-driven oxygen consumption is altered in *mdx* mice. In order to determine whether the decrease in the respiration mediated by complex I is related to Ca^2+^, we measured complex I-driven oxygen consumption in the presence of 400 nM Ca^2+^. In this condition, the complex I-mediated respiration rate was significantly enhanced in *mdx* mitochondria compared to WT (Figure 4B), while the expression of complex I subunits is unchanged (Figures 4C,D). It is noteworthy that the expression of the other complexes is also unchanged compared to WT (Supplementary Figure 6). Another parameter which could explain the decrease in complex I-driven oxygen consumption is the capacity of the complex I to transfer electron. We performed quantification of enzymatic activity, but we did not observe any modification in enzymatic activities of complexes I and IV nor of the activity of citrate synthase in *mdx* mice (Figure 4E). A preserved complex I activity and a reduced complex I-mediated respiration could indicate an electron leak at the complex I level and superoxide anion (O_2_.−) production. We have indeed observed an increase in Mitosox red fluorescence in paced *mdx* cardiomyocytes but also in the presence of the complex III inhibitor antimycin A, demonstrating an increase in the production of O_2_.− at the level of complex I compared to WT (Figure 4F). Altogether, these data indicate an impaired carbohydrate-mediated mitochondrial respiration at the level of complex I which is partly compensated by the increase in mitochondrial Ca^2+^.

### Metformin Improves SR/ER-Mitochondrial Interaction and Mitochondrial Function

**Figure 5:**
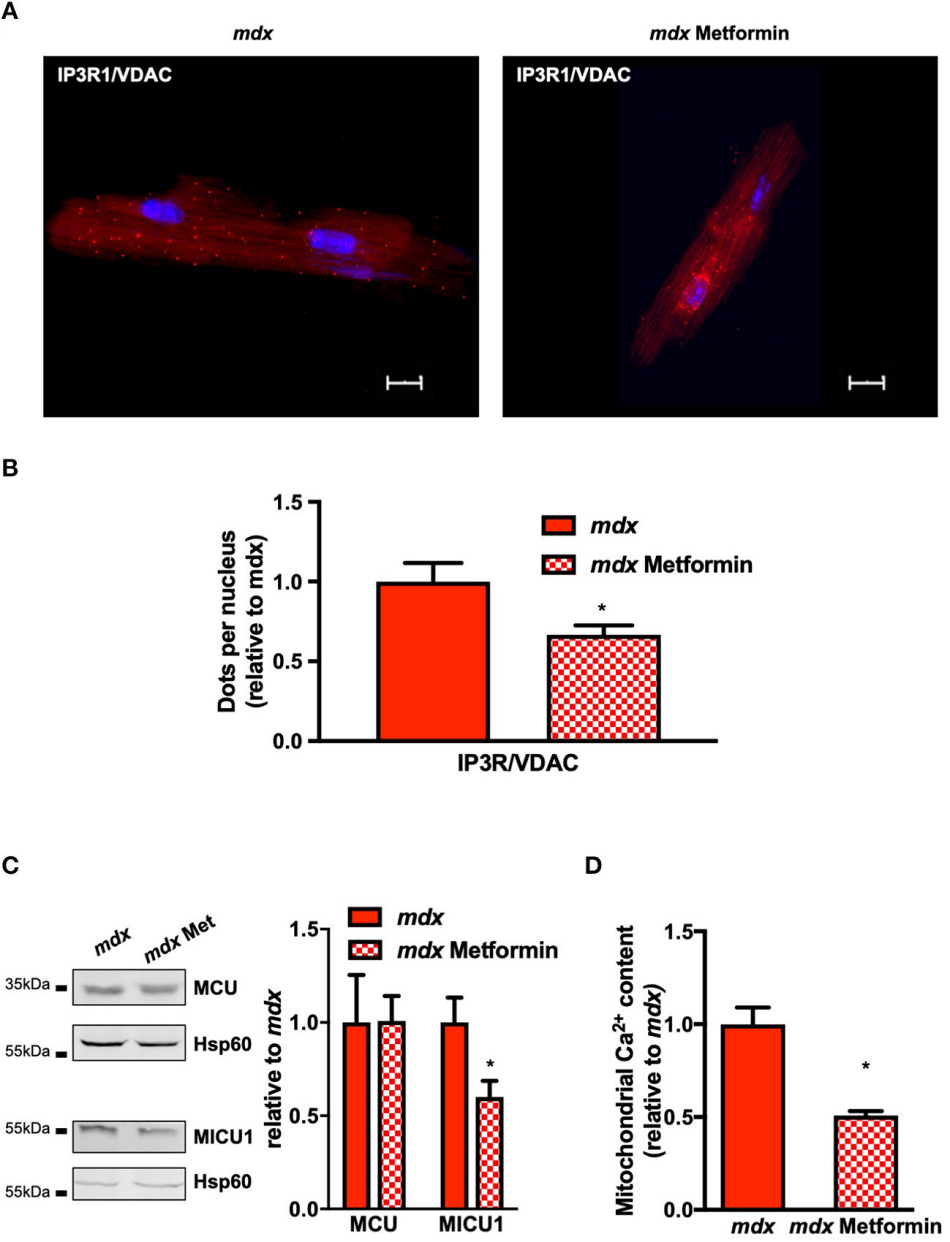
Chronic metformin treatment decreases SR/ER and mitochondria interaction and mitochondrial Ca^2+^ content in *mdx* cardiomyocytes. All Western blots were performed on isolated mitochondria, and quantifications of proteins were normalized to Hsp60 and expressed relative to *mdx*. **(A, B)** Representative images and quantitative analysis of IP3R1–VDAC interaction measured by *in situ* PLA on isolated cardiomyocytes from *mdx* (*N* = 3, *n* = 49) and *mdx* + metformin (*N* = 2; *n* = 33). The numbers of dots were normalized to the number of nucleus per cells and expressed relative to *mdx* mean value. Mean ± SEM, *p = 0.046 *mdx* vs. *mdx* + metformin. **(C)** Representative immunoblots and quantification of MCU and MICU1. Data are mean ± SEM, *p = 0.0303 *mdx* (*N* = 6) vs. *mdx* + metformin (*N* = 6). **(D)** Mean of the absolute mitochondrial Ca^2+^ content in *mdx* (*N* = 4) and *mdx* + metformin (*N* = 6) hearts. Mean ± SEM, *p = 0.0095 mdx vs. *mdx* + metformin.

**Figure 6:**
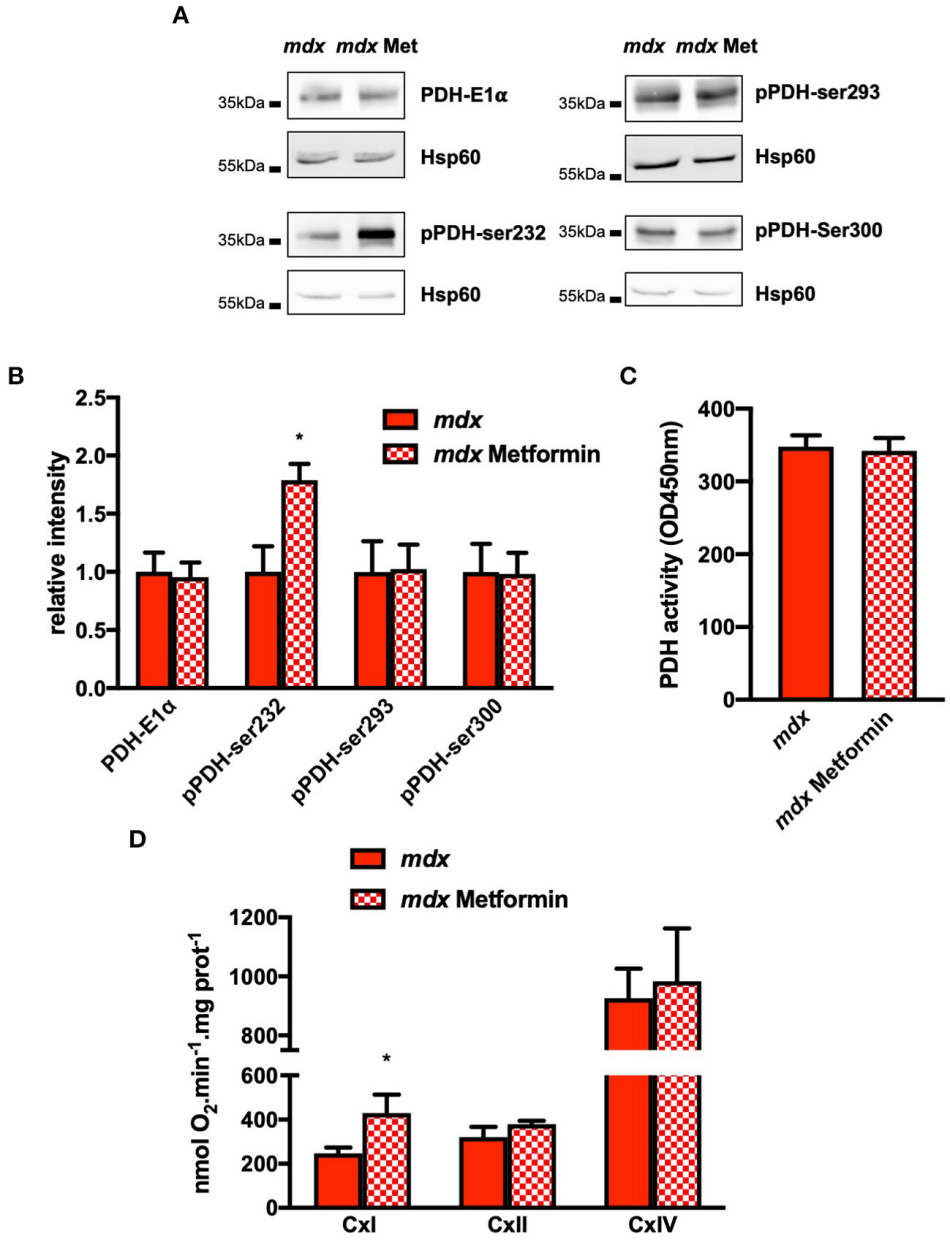
Chronic metformin treatment increases PDH Ser232 phosphorylation and complex I-mediated respiration in *mdx* hearts. All Western blots were performed on isolated mitochondria and quantifications of proteins were normalized to Hsp60 and expressed relative to *mdx*. **(A)** Representative immunoblots and **(B)** quantification of total pyruvate dehydrogenase subunit E1α (PDH-E1α), PDH phosphorylated on Serine 232, 293, and 300. Data are mean ± SEM, *p = 0.0317 *mdx* (*N* = 4–6) vs. *mdx* + metformin (*N* = 4–6). **(C)** Mean PDH activity measured on isolated mitochondria. Data are mean ± SEM, *p* > 0.05 *mdx* (*N* = 6) vs. *mdx* + metformin (*N* = 7). **(D)** Complex I-dependent respiration State 3 (EIII) is determined in the presence of malate and pyruvate (CxI). Complex II- and IV-dependent respiration State 3 (EIII) is obtained by addition of succinate and rotenone (CxII) and ascorbate/TMPD (CxIV), respectively. Data are mean ± SEM, *p = 0.036 *mdx* (*N* = 6) vs. *mdx* + metformin (*N* = 9).

We next investigated the effects of a treatment with metformin, the antidiabetic drugs targeting mitochondrial complex I. After 1 month in drinking water, the level of phosphorylation of AMPK and acetyl-CoA carboxylase (ACC), one of the downstream targets of AMPK, was significantly increased validating the efficacy treatment with metformin (Supplementary Figure 7). We next evaluated the SR/ER–mitochondria interaction using *in situ* PLA assay. As shown in Figure 5, metformin treatment decreased IP3R1/VDAC contacts (Figures 5A,B) whereas the expression levels of IP3R1, VDAC, GRP75, or Sig-1R remained similar (Supplementary Figure 7). Although MCU expression is also unchanged, MICU1 level is decreased and this is accompanied by a decrease in the mitochondrial Ca^2+^ content (Figures 5C,D). The decrease in mitochondrial Ca^2+^ content is accompanied by an increase of the phosphorylation level of the PDH but only on the Ser232 site (Figures 6A,B). However, this is not sufficient to affect the PDH activity which remained comparable to untreated *mdx* mice (Figure 6C). Finally, the complex I-mediated oxygen consumption under pyruvate/malate substrate is significantly enhanced (Figure 6D), indicating that metformin treatment in addition to restoration of SR/ER-mitochondrial interactions optimizes mitochondrial function in *mdx* ventricular cardiomyocytes.

## Discussion

Dystrophin deficiency causes profound striated skeletal muscle lesions which lead to major muscle weakness at the early stage of the pathology and ultimately to fatal respiratory failure. Secondary to peripheral muscle deficiencies, a progressive cardiomyopathy develops with left ventricle dilatation, fibrosis, and arrhythmias (Sasaki et al., 1998; Fauconnier et al., 2010; Amedro et al., 2019; Segawa et al., 2020). Over the last decade, due to the improvement of the management of patients' respiratory failure, cardiac failure has become a major cause of death. Although cardiac and skeletal muscles share similar pathophysiological mechanisms, which are more or less shifted in time, some of these processes appear to be regulated differently. Indeed, despite an increase of IP3R1 expression in both tissues, here we demonstrated an increase in SR/ER–mitochondria interactions, characterized by an increase in IP3R1–GRP75–VDAC contact points, whereas in skeletal fibers such interactions were decreased (Pauly et al., 2017). Interestingly, the IP3R1/GRP75 or IP3R1/VDAC1 protein–protein binding remained unchanged, indicating that the elevation in PLA signals is most likely related to an increase in IP3R1 expression. Although the expression of MFN2 is unchanged, electron microscopy would be useful to establish whether the SR/ER–mitochondria tethering, and the physical distances between the two organelles, is disturbed in mdx hearts prior to the development of DCM. IP3Rs are involved in many cellular processes like metabolism, secretion, gene transcription, cell fate, and ER stress (Kiviluoto et al., 2013), but compared to RyR2 which is 50–100 times more expressed in left ventricles, the contribution of the IP3Rs to the cardiac beat-to-beat Ca^2+^ homeostasis and SR Ca^2+^ leak is unlikely (Kockskämper et al., 2008).

Increase of IP3R1 expression is accompanied by an elevation in Sig-1R, a transmembrane chaperone located in MAMs interacting with IP3Rs and ER stress sensors (for review Delprat et al., 2020). Sig-1R has been shown to (i) stabilize IP3R1 in MAMs contributing to the strengthening of ER–mitochondria contact and ER–mitochondria Ca^2+^ transfer and (ii) bind the protein chaperone BiP in the ER lumen stabilizing the ER stress response proteins (Hayashi and Su, 2007). Although a prolonged disrupted IP3R1–GRP75–VDAC interaction promotes ER stress (Rieusset et al., 2016; Pauly et al., 2017), an increase in contact points and Sig-1R expression has been reported in the early stage of the UPR response to sustain cell homeostasis and bioenergetics and alleviate ER stress (Hayashi and Su, 2007; Bravo et al., 2011; Delprat et al., 2020). Consistently, in contrast to skeletal muscle and with the exception of *Atf3*, UPR-inducible genes and ER sensor expression remained unchanged in the present study. Of note, the absence of ER stress response in the *mdx* heart does not exclude a mitochondrial stress response *per se*.

As indicated, the mitigation of ER stress might be related to an enhancement of Ca^2+^ transfer and mitochondrial function (Bravo et al., 2011). Here, the increase in the IP3R1–GRP75–VDAC1 juxtaposition was associated with an increase in the MCU–MICU1 complex suggesting a reinforcement of the IP3R1–GRP75–VDAC–MCU Ca^2+^ transfer axis. As shown recently by Dubinin et al. (2020), we also observed an increase expression of MICU1. MICU1/2 subunits form heterodimers located in the mitochondrial intermembrane space regulating the Ca^2+^-dependent gating and threshold properties of the MCU. At low Ca^2+^ concentrations, the MICU1/MICU2 dimer keeps MCU in the close state, and at higher Ca^2+^ level, MICU2-dependent inhibition is released and MICU1 activates MCU allosterically (Payne et al., 2017; Tarasova et al., 2019). An increase in the MICU1/MICU2 ratio reduces the Ca^2+^ threshold for MCU activation and increases the Ca^2+^ sensitivity of the MCU complex (Payne et al., 2017). Here, increased expression of MCU and MICU1 is not associated with a change in MICU2, which may in addition to decreased expression of the dominant negative isoform of MCU (MCUb) enhanced the mitochondrial Ca^2+^ uptake and content as shown in *mdx* ventricular mitochondria [Figure 2; (Dubinin et al., 2020)]. This change in MICU1 expression may affect the pharmacology of MCU and should be considered for future therapeutic strategies aimed at directly targeting mitochondrial Ca^2+^ uptake (Kon et al., 2017; Márta et al., 2020). The elevation of mitochondrial Ca^2+^ content is therefore the result of a remodeling of the MCU complex associated with the increase in the leakage of Ca^2+^ from the SR, the elevation of diastolic Ca^2+^, and the strengthening of the contact points between the two organelles.

More generally, the enhancement of mitochondrial Ca^2+^ uptake and content has several functional consequences. It increases the metabolic and respiration rate by stimulating the respiratory chain and the activities of several enzymes involved in the metabolic flux and the Krebs cycle. Among them, the PDH is the entry point for the glycolytic product pyruvate into the oxidative metabolism. In the mitochondrial matrix, Ca^2+^ activates the PDH phosphatase 1 that dephosphorylates PDH to increase its activity, and thus the use of carbohydrates for energy production. Here PDH phosphorylation at three serine residues (pSer232, pSer293, pSer300 in the mouse) on the alpha chain of the E1 subunit is significantly decreased in conjunction with an increase in PDH activity. This increases in PDH activity may account for the previously described elevation in the pyruvate decarboxylation and the shift from fatty acid to carbohydrate oxidation in the heart of both *mdx* and DMD patients (Perloff et al., 1984; Quinlivan et al., 1996; Momose et al., 2001; Naruse et al., 2004; Khairallah et al., 2007). PDH catalyzes the irreversible step of oxidative decarboxylation of pyruvate to produce acetyl-CoA and fuel the tricarboxylic acid cycle and electron transport chain. The increased PDH activity thus enhances the glycolytic flux, and pyruvate becomes the privileged substrate for the oxidative phosphorylation (Sun et al., 2015). However, in the absence of Ca^2+^ and as recently reported, the pyruvate-driven complex I respiration is reduced independently of any change in complex I expression level (Hughes et al., 2020). Such impairment might be related to posttranslational modifications; however, intrinsic complex I activity remained unchanged. It is noteworthy that mitochondrial respiration analyzed with substrates other than pyruvate, such as glutamate, does not appear to show any difference in *mdx* heart (Ascah et al., 2011; Viola et al., 2013). Remarkably, in the *mdx* diaphragm, the mitochondrial rate of ATP production was partly improved by directly stimulating Complex II, suggesting that Kreb's-driven NADH-dependent complex I function is defective (Rybalka et al., 2014). In addition, in the presence of Ca^2+^, complex I-mediated mitochondrial respiration is boosted, suggesting adaptive mechanisms to sustain the energy demand. On the one hand, it can increase the rate of pyruvate consumption to improve NADH production but, on the other hand, enhance electron leakage and mitochondrial ROS production (Williams and Allen, 2007b; Viola et al., 2013). Although an increase in mitochondrial ROS production is commonly observed in the heart of *mdx* (Williams and Allen, 2007b; Viola et al., 2013; Kuno et al., 2018; Hughes et al., 2020), the exact mechanisms linking complex I to ROS production remain to be established. It would be interesting to determine whether ROS are produced in the forward direction or in the reverse direction of electron transfer (Hirst and Roessler, 2016). Although we have not explored these mechanisms in detail, electron leakage from complex II is somewhat unlikely because the succinate-induced respiration is comparable in *mdx* and WT hearts. In the forward mode hypothesis, targeting the function of complex I may improve mitochondrial activity and metabolism.

We here tested metformin, an antidiabetic drug with pleiotropic properties that are related to its mitochondrial effects (for review see Foretz et al., 2014; Vial et al., 2019). Long-term treatment with metformin has already demonstrated some beneficial effects on motor function in stable DMD patients with encouraging evidence regarding muscle degeneration and histopathology (Hafner et al., 2016, 2019). The exact mechanisms of action of metformin have yet to be elucidated; however, at high doses, metformin inhibits the oxidation of NADH at the complex I level. Consequently, the ADP:ATP or AMP:ATP ratios increase which is thought to activate AMPK, a hub for major metabolic and energy sensing pathways (Horman et al., 2012; Foretz et al., 2014; He and Wondisford, 2015). In addition, at low doses, metformin has also been shown to activate AMPK independent of direct inhibition of complex I (He and Wondisford, 2015). In all cases, activation of AMPK demonstrated significant beneficial effects on the dystrophic phenotype in skeletal and cardiac muscles. Importantly, it improves mechanical signaling, muscle strength, and force; limits muscle necrosis, fibrosis, and inflammation; and stimulates the oxidative phenotype, mitochondrial function, and autophagy processes (Ljubicic et al., 2011, 2012; Pauly et al., 2012; Garbincius and Michele, 2015; Juban et al., 2018). Metformin has also been shown to increase PGC1-α expression in *mdx* muscle fibers, a central transcriptional coactivator regulating a wide range of biological processes such as mitochondrial biogenesis, oxidative phosphorylation, and muscle regeneration (Scarpulla, 2011; Ljubicic and Jasmin, 2015; Suntar et al., 2020). In addition, increased activation of AMPK stimulates autophagic clearance of defective mitochondria and may thus improve mitochondrial function (De Palma et al., 2012; Pauly et al., 2012). More generally, the maintenance of cell fate and cell proteostasis are emerging therapeutic targets in DMD (De Palma et al., 2012), but depending on the phase, severity, and inflammatory state of the pathology, the therapeutic window is critical for such emerging strategies (Farini et al., 2019). Here, in line with other studies, treatment with metformin also improves pyruvate-mediated mitochondrial respiration (Wang et al., 2019) and increasing the level of ACC phosphorylation would also facilitate fatty acid oxidation (Saddik et al., 1993). In parallel, MICU1 expression decreased but MCU did not, which is consistent with a recent report demonstrating a causal link between the pyruvate fluxe and consumption and MICU1 expression, suggesting that MICU1 could serve as a metabolic sensor (Nemani et al., 2020). Moreover, the IP3R1–VDAC1 interactions also decrease, indicating a reduction in the SR/ER–mitochondria contact points. Although the mechanism remains to be established, changes in SR/ER–mitochondria interaction are causally related to energy metabolism. AMPK activation has recently been shown to reduce the formation of cardiac MAM in hyperglycemia and decrease IP3-induced Ca^2+^ release (Arias-del-Val et al., 2019; Wu et al., 2019). Remodeling of the IP3R1–VDAC1–MCU axis under metformin treatment also reduced mitochondrial Ca^2+^ content and enhanced the phosphorylation of the Ser232 site of the PDH-E1α subunit, which was not sufficient to reduce PDH activity in *mdx* cardiomyocytes. All three phosphorylation sites can restrain enzyme activity; however, Ser293 phosphorylation has a higher inhibitory impact than Ser300 and even more than Ser232 phosphorylation (Korotchkina and Patel, 1995; Gray et al., 2014). Finally, metformin can also impact ROS production in a different way, first of all as a modulator of complex I activity and then by its effect on mitochondrial Ca^2+^ content. AMPK activation has also been reported to decrease mitochondrial production of ROS (Foretz et al., 2014).

Myocardial metabolic and mitochondrial impairments as well as mitochondrial and cellular Ca^2+^ handling have been extensively studied in DMD during the last decade. All the studies agree that these disorders, which precede the onset of structural remodeling and deterioration of myocardial macroscopic function, play a central role in the progression of the pathology to heart failure (for review: Esposito and Carsana, 2019). Here, for the first time, our data suggests that the enhancement of SR/ER–mitochondria contact points and increasing mitochondrial Ca^2+^ uptake and content would enhance glycolytic flux and complex I respiration. However, the downside of this scheme is that increasing mitochondrial Ca^2+^ would increase ROS production, sensitize mPTP, further impair metabolic flexibility, and alter ATP production and Ca^2+^ handling creating an amplification loop with all the ingredients toward contractile dysfunction, arrhythmia, fibrosis, and finally heart failure. Accordingly, long-term metformin treatment has already proved to have some beneficial effects on the development of DCM linked to mutations in the dystrophin–glycoprotein complex (Mantuano et al., 2018; Kanamori et al., 2019). Although more studies are needed to understand and characterize the beneficial effects of chronic treatment with metformin on the development of the cardiomyopathy associated with DMD, in the present study, metformin already demonstrates beneficial effects on the aberrant SR/ER-mitochondria interaction and increased mitochondrial Ca^2+^ as well as mitochondrial function. It will therefore be very important in the near future to determine whether treatment with metformin can improve dystrophic cardiomyopathy in the advanced stage of the pathology. To conclude, our data support the concept that metformin alone or in combination with other drugs might be a potential therapeutic strategy to ameliorate the dystrophinopathies (Casteels et al., 2010; Ljubicic and Jasmin, 2015; Hafner et al., 2016, 2019; Mantuano et al., 2018; Vitiello et al., 2019).

## Data Availability Statement

The original contributions presented in the study are included in the article/Supplementary Material, further inquiries can be directed to the corresponding author/s.

## Ethics Statement

The animal study was reviewed and approved by Comité d'éthique régional en expérimentation animale languedoc Roussillon–Ministère de l'enseignement supérieur de la recherche et de l'innovation (N° #16473-2018082016141320).

## Author Contributions

CA and MP performed and analyzed the experiments. ML and JR performed the experiments. AL designed the experiments, discussed the data, and read the manuscript. JF designed the experiments, collected and discussed the data, and wrote the manuscript. All authors contributed to the article and approved the submitted version.

## Conflict of Interest

The authors declare that the research was conducted in the absence of any commercial or financial relationships that could be construed as a potential conflict of interest.

## Supplementary Material

Supplementary Figure 1 Respiratory control ratio (RCR) was measured in **(A)** WT (*N* = 7), *mdx* (*N* = 13) and *mdx* + met (*N* = 9) hearts and **(B)** in WT (*N* = 6) and *mdx* (*N* = 6) in presence of in presence of 0 nM or 400 nM extramitochondrial Ca^2+^. Data are mean ± SEM, *p* > 0.05 *mdx* vs. WT.

Supplementary Figure 2 SERCA2a, PLB expression, and SR Ca^2+^ load. **(A)** Full length immunoblots and quantification of SERCA2a, phospholamban (PLB), and Phospho-Phospholamban (Ser16/Thr17) (pPLB-Ser16; pPLB-Thr17) were normalized to GAPDH. Data are mean ± SEM, *p* > 0.05 *mdx* (*N* = 4-6) *vs*. WT (*N* = 4-6). **(B)** Mean values of the amplitude of caffeine-induced SR Ca^2+^ release, estimating the SR Ca^2+^ load. ^*^*p* = 0.0182 WT (*N* = 3; *N* = 9) vs. *mdx* (*N* = 3; *N* = 10).

Supplementary Figure 3 IP3R1 immunoprecipitation. Anti-IP3R1 antibody (1:200) was used to immunoprecipitate IP3R1 from heart homogenate. Samples were incubated with an anti-IP3R1 antibody in 0.5 ml of a modified RIPA buffer (10 mM Tris–HCl, pH 7.4; 150 mM NaCl; 1% Triton; 5 mM NaF and protease inhibitor cocktail) for 2 h at 4°C. The immune complex was incubated with protein A/G magnetic beads (Pierce 88802) at 4°C for 2 h, after which the beads were washed out three times with RIPA buffer. Proteins were separated on SDS/PAGE gels and transferred onto nitrocellulose membranes for 1 h at 100 V. The immunoblots were prepared using antibodies against IP3R1 (1:1,000), anti-GRP75 antibody (1:1,000, Santa Cruz) and anti-VDAC (1:300). All immunoblots were developed and quantified using the Odyssey infrared imaging system (LICOR Biosystems) and infrared-labeled secondary antibodies. After immunoprecipitation (IP) of IP3R1 from heart muscle of WT or mdx mice, immunoblots were used to detect IP3R1, Grp75, and VDAC. Data are mean ± SEM, *p* > 0.05 *mdx* (*N* = 3) vs. WT (*N* = 3).

Supplementary Figure 4 **(A)** Transcript levels of UPR response genes quantified by 30 reverse transcription quantitative polymerase chain reaction (RT-qPCR) in WT (*N* = 4) and *mdx* (*N* = 4) hearts. The mRNA levels were normalized to the reference gene TBP. Data are means ± SEM, ^*^*p* = 0.0286 *mdx* vs. WT. **(B)** PLA technical negative control obtained in absence of primary antibody.

Supplementary Figure 5 Pyruvate dehydrogenase kinase 4 (PDK4) expression. Full length immunoblots and quantification of PDK4 were normalized to Hsp60. Data are mean ± SEM, *p* > 0.05 *mdx* (*N* = 5) vs. WT (*N* = 5).

Supplementary Figure 6 Protein expression of mitochondrial respiratory chain complexes. All western blots were performed on isolated mitochondria and quantifications of proteins are normalized to Hsp60. **(A)** Representative immunoblots and **(B)** quantification of complex I (CxI), complex 40 II(CxII), complex III (CxIII), complex IV (CxIV), complex V (Cx V). Data are mean ± SEM, *mdx* (*N* = 6) vs. WT (*N* = 6).

Supplementary Figure 7 Phosphorylation level of AMPK and ACC, and proteins expression of SR/ER and mitochondria contacts points. All quantifications of proteins are normalized either to GAPDH or Actin when heart homogenates were used or to Hsp60 when western blot were performed on isolated mitochondria. **(A)** Representative immunoblots and quantification of IP3R1, GRP75, Sig-1R, and VDAC. Quantification of IP3R1, GRP75, MFN2, and Sig-1R was carried out from *N* = 6 *mdx* and *mdx* +metformin hearts. Data are mean ± SEM. **(B)** Mean value of pAMPK/AMPK and pACC/ACC ratio. Ratio were established after proteins normalization to Actin from *N* = 6 WT, *mdx* and *mdx* +metformin hearts. Full lengths gels are in supplemental information. WT vs. *mdx*+ metformin ^#^*p* = 0.0101 and *mdx* vs. *mdx*+metformin ^*^*p* = 0.0419.

Supplementary Information All full lengths gel for all protein probed.

## References

[B1] Amedro P. Vincenti M. De La Villeon G. Lavastre K. Barrea C. Guillaumont S. (2019). Speckle-tracking echocardiography in children with Duchenne muscular dystrophy: a prospective multicenter controlled cross-sectional study. J. Am. Soc. Echocardiogr. Off. Publ. Am. Soc. Echocardiogr. 32, 412–422. 10.1016/j.echo.2018.10.017 30679141

[B2] Angebault C. Fauconnier J. Patergnani S. Rieusset J. Danese A. Affortit C. A.. (2018). ER-mitochondria cross-talk is regulated by the Ca2+ sensor NCS1 and is impaired in Wolfram syndrome. Sci. Signal. 11:eaaq1380. 10.1126/scisignal.aaq1380 30352948

[B3] Arias-del-Val J. Santo-Domingo J. García-Casas P. Alvarez-Illera P. Núñez Galindo A. Wiederkehr A.. (2019). Regulation of inositol 1,4,5-trisphosphate-induced Ca2+ release from the endoplasmic reticulum by AMP-activated kinase modulators. Cell Calcium 77, 68–76. 10.1016/j.ceca.2018.12.004 30557841

[B4] Ascah A. Khairallah M. Daussin F. Bourcier-Lucas C. Godin R. Allen B. G.. (2011). Stress-induced opening of the permeability transition pore in the dystrophin-deficient heart is attenuated by acute treatment with sildenafil. Am. J. Physiol. Heart Circ. Physiol. 300, H144–H153. 10.1152/ajpheart.00522.2010 20971771

[B5] Bravo R. Vicencio J. M. Parra V. Troncoso R. Munoz J. P. Bui M.. (2011). Increased ER-mitochondrial coupling promotes mitochondrial respiration and bioenergetics during early phases of ER stress. J. Cell Sci. 124, 2143–2152. 10.1242/jcs.080762 21628424 PMC3113668

[B6] Burelle Y. Khairallah M. Ascah A. Allen B. G. Deschepper C. F. Petrof B. J.. (2010). Alterations in mitochondrial function as a harbinger of cardiomyopathy: lessons from the dystrophic heart. J. Mol. Cell. Cardiol. 48, 310–321. 10.1016/j.yjmcc.2009.09.004 19769982 PMC5298900

[B7] Casteels K. Fieuws S. van Helvoirt M. Verpoorten C. Goemans N. Coudyzer W. (2010). Metformin therapy to reduce weight gain and visceral adiposity in children and adolescents with neurogenic or myogenic motor deficit. Pediatr. Diabetes 11, 61–69. 10.1111/j.1399-5448.2009.00512.x 19496972

[B8] De Palma C. Morisi F. Cheli S. Pambianco S. Cappello V. Vezzoli M.. (2012). Autophagy as a new therapeutic target in Duchenne muscular dystrophy. Cell Death Dis. 3:e418. 10.1038/cddis.2012.159 23152054 PMC3542595

[B9] Delprat B. Crouzier L. Su T.-P. Maurice T. (2020). At the crossing of ER stress and MAMs: a key role of sigma-1 receptor? Adv. Exp. Med. Biol. 1131, 699–718. 10.1007/978-3-030-12457-1_28 31646531

[B10] Dubinin M. V. Talanov E. Yu Tenkov K. S. Starinets V. S. Mikheeva I. B.. (2020). Transport of Ca2+ and Ca2+-dependent permeability transition in heart mitochondria in the early stages of Duchenne muscular dystrophy. Biochim. Biophys. Acta Bioenerg. 1861:148250. 10.1016/j.bbabio.2020.148250 32569663

[B11] Esposito G. Carsana A. (2019). Metabolic alterations in cardiomyocytes of patients with Duchenne and Becker muscular dystrophies. J. Clin. Med. 8:2151. 10.3390/jcm8122151 31817415 PMC6947625

[B12] Farini A. Gowran A. Bella P. Sitzia C. Scopece A. Castiglioni E.. (2019). Fibrosis rescue improves cardiac function in dystrophin-deficient mice and Duchenne patient–specific cardiomyocytes by immunoproteasome modulation. Am. J. Pathol. 189, 339–353. 10.1016/j.ajpath.2018.10.010 30448404

[B13] Fauconnier J. Andersson D. C. Zhang S.-J. Lanner J. T. Wibom R. Katz A.. (2007). Effects of palmitate on Ca(2+) handling in adult control and ob/ob cardiomyocytes: impact of mitochondrial reactive oxygen species. Diabetes 56, 1136–1142. 10.2337/db06-0739 17229941

[B14] Fauconnier J. Lanner J. T. Zhang S.-J. Tavi P. Bruton J. D. Katz A.. (2005). Insulin and inositol 1,4,5-trisphosphate trigger abnormal cytosolic Ca2+ transients and reveal mitochondrial Ca2+ handling defects in cardiomyocytes of ob/ob mice. Diabetes 54, 2375–2381. 10.2337/diabetes.54.8.2375 16046304

[B15] Fauconnier J. Thireau J. Reiken S. Cassan C. Richard S. Matecki S.. (2010). Leaky RyR2 trigger ventricular arrhythmias in Duchenne muscular dystrophy. Proc. Natl. Acad. Sci. U.S.A. 107, 1559–1564. 10.1073/pnas.0908540107 20080623 PMC2824377

[B16] Foretz M. Guigas B. Bertrand L. Pollak M. Viollet B. (2014). Metformin: from mechanisms of action to therapies. Cell Metab. 20, 953–966. 10.1016/j.cmet.2014.09.018 25456737

[B17] Frezza C. Cipolat S. Scorrano L. (2007). Organelle isolation: functional mitochondria from mouse liver, muscle, and cultured fibroblasts. Nat. Protoc. 2, 287–295. 10.1038/nprot.2006.478 17406588

[B18] Garbincius J. F. Michele D. E. (2015). Dystrophin-glycoprotein complex regulates muscle nitric oxide production through mechanoregulation of AMPK signaling. Proc. Natl. Acad. Sci. U.S.A. 112, 13663–13668. 10.1073/pnas.1512991112 26483453 PMC4640723

[B19] Gray L. R. Tompkins S. C. Taylor E. B. (2014). Regulation of pyruvate metabolism and human disease. Cell. Mol. Life Sci. 71, 2577–2604. 10.1007/s00018-013-1539-2 24363178 PMC4059968

[B20] Hafner P. Bonati U. Erne B. Schmid M. Rubino D. Pohlman U.. (2016). Improved muscle function in Duchenne muscular dystrophy through L-arginine and metformin: an investigator-initiated, open-label, single-center, proof-of-concept-study. PLoS ONE 11:e0147634. 10.1371/journal.pone.0147634 26799743 PMC4723144

[B21] Hafner P. Bonati U. Klein A. Rubino D. Gocheva V. Schmidt S.. (2019). Effect of combination l-citrulline and metformin treatment on motor function in patients with Duchenne muscular dystrophy: a randomized clinical trial. JAMA Netw. Open 2:e1914171. 10.1001/jamanetworkopen.2019.14171 31664444 PMC6824222

[B22] Hayashi T. Su T.-P. (2007). Sigma-1 receptor chaperones at the ER- mitochondrion interface regulate Ca2+ signaling and cell survival. Cell 131, 596–610. 10.1016/j.cell.2007.08.036 17981125

[B23] He L. Wondisford F. E. (2015). Metformin action: concentrations matter. Cell Metab. 21, 159–162. 10.1016/j.cmet.2015.01.003 25651170

[B24] Hirst J. Roessler M. M. (2016). Energy conversion, redox catalysis, and generation of reactive oxygen species by respiratory complex I. Biochim. Biophys. Acta Bioenerg. 1857, 872–883. 10.1016/j.bbabio.2015.12.009 26721206 PMC4893023

[B25] Hoffman E. P. (2020). The discovery of dystrophin, the protein product of the Duchenne muscular dystrophy gene. FEBS J. 287, 3879–3887. 10.1111/febs.15466 32608079 PMC7540009

[B26] Horman S. Beauloye C. Vanoverschelde J.-L. Bertrand L. (2012). AMP-activated protein kinase in the control of cardiac metabolism and remodeling. Curr. Heart Fail. Rep. 9, 164–173. 10.1007/s11897-012-0102-z 22767403

[B27] Hughes M. C. Ramos S. V. Turnbull P. C. Edgett B. A. Huber J. S. Polidovitch N.. (2020). Impairments in left ventricular mitochondrial bioenergetics precede overt cardiac dysfunction and remodelling in Duchenne muscular dystrophy. J. Physiol. 598, 1377–1392. 10.1113/JP277306 30674086

[B28] Juban G. Saclier M. Yacoub-Youssef H. Kernou A. Arnold L. Boisson C.. (2018). AMPK activation regulates LTBP4-dependent TGF-β1 secretion by pro-inflammatory macrophages and controls fibrosis in Duchenne muscular dystrophy. Cell Rep. 25, 2163–2176. 10.1016/j.celrep.2018.10.077 30463013

[B29] Jung C. Martins A. S. Niggli E. Shirokova N. (2008). Dystrophic cardiomyopathy: amplification of cellular damage by Ca2+ signalling and reactive oxygen species-generating pathways. Cardiovasc. Res. 77, 766–773. 10.1093/cvr/cvm089 18056762

[B30] Kanamori H. Naruse G. Yoshida A. Minatoguchi S. Watanabe T. Kawaguchi T.. (2019). Metformin enhances autophagy and provides cardioprotection in δ-sarcoglycan deficiency-induced dilated cardiomyopathy. Circ. Heart Fail. 12:e005418. 10.1161/CIRCHEARTFAILURE.118.005418 30922066

[B31] Khairallah M. Khairallah R. Young M. E. Dyck J. R. B. Petrof B. J. Des Rosiers C. (2007). Metabolic and signaling alterations in dystrophin-deficient hearts precede overt cardiomyopathy. J. Mol. Cell. Cardiol. 43, 119–129. 10.1016/j.yjmcc.2007.05.015 17583724

[B32] Khairallah M. Khairallah R. J. Young M. E. Allen B. G. Gillis M. A. Danialou G.. (2008). Sildenafil and cardiomyocyte-specific cGMP signaling prevent cardiomyopathic changes associated with dystrophin deficiency. Proc. Natl. Acad. Sci. U.S.A. 105, 7028–7033. 10.1073/pnas.0710595105 18474859 PMC2383977

[B33] Kiviluoto S. Vervliet T. Ivanova H. Decuypere J.-P. De Smedt H. Missiaen L.. (2013). Regulation of inositol 1,4,5-trisphosphate receptors during endoplasmic reticulum stress. Biochim. Biophys. Acta 1833, 1612–1624. 10.1016/j.bbamcr.2013.01.026 23380704

[B34] Kockskämper J. Zima A. V. Roderick H. L. Pieske B. Blatter L. A. Bootman M. D. (2008). Emerging roles of inositol 1,4,5-trisphosphate signaling in cardiac myocytes. J. Mol. Cell. Cardiol. 45, 128–147. 10.1016/j.yjmcc.2008.05.014 18603259 PMC2654363

[B35] Kon N. Murakoshi M. Isobe A. Kagechika K. Miyoshi N. Nagayama T. (2017). DS16570511 is a small-molecule inhibitor of the mitochondrial calcium uniporter. Cell Death Discov. 3:17045. 10.1038/cddiscovery.2017.45 28725491 PMC5511861

[B36] Korotchkina L. G. Patel M. S. (1995). Mutagenesis studies of the phosphorylation sites of recombinant human pyruvate dehydrogenase. Site-specific regulation. J. Biol. Chem. 270, 14297–14304. 10.1074/jbc.270.24.14297 7782287

[B37] Kuno A. Hosoda R. Sebori R. Hayashi T. Sakuragi H. Tanabe M.. (2018). Resveratrol ameliorates mitophagy disturbance and improves cardiac pathophysiology of dystrophin-deficient mdx mice. Sci. Rep. 8:15555. 10.1038/s41598-018-33930-w 30348945 PMC6197260

[B38] Kuznetsov A. V. Winkler K. Wiedemann F. R. von Bossanyi P. Dietzmann K. Kunz W. S. (1998). Impaired mitochondrial oxidative phosphorylation in skeletal muscle of the dystrophin-deficient mdx mouse. Mol. Cell. Biochem. 183, 87–96. 10.1023/A:1006868130002 9655182

[B39] Kyrychenko V. Poláková E. Janíček R. Shirokova N. (2015). Mitochondrial dysfunctions during progression of dystrophic cardiomyopathy. Cell Calcium 58, 186–195. 10.1016/j.ceca.2015.04.006 25975620 PMC4501876

[B40] Lee S. Min K.-T. (2018). The interface between ER and mitochondria: molecular compositions and functions. Mol. Cells 41, 1000–1007. 10.14348/molcells.2018.0438 30590907 PMC6315321

[B41] Ljubicic V. Jasmin B. J. (2015). Metformin increases peroxisome proliferator-activated receptor γ Co-activator-1α and utrophin a expression in dystrophic skeletal muscle. Muscle Nerve 52, 139–142. 10.1002/mus.24692 25908446

[B42] Ljubicic V. Khogali S. Renaud J.-M. Jasmin B. J. (2012). Chronic AMPK stimulation attenuates adaptive signaling in dystrophic skeletal muscle. Am. J. Physiol. Cell Physiol. 302, C110–C121. 10.1152/ajpcell.00183.2011 21940670

[B43] Ljubicic V. Miura P. Burt M. Boudreault L. Khogali S. Lunde J. A.. (2011). Chronic AMPK activation evokes the slow, oxidative myogenic program, and triggers beneficial adaptations in mdx mouse skeletal muscle. Hum. Mol. Genet. 20, 3478–3493. 10.1093/hmg/ddr265 21659335

[B44] Mantuano P. Sanarica F. Conte E. Morgese M. G. Capogrosso R. F. Cozzoli A.. (2018). Effect of a long-term treatment with metformin in dystrophic mdx mice: a reconsideration of its potential clinical interest in Duchenne muscular dystrophy. Biochem. Pharmacol. 154, 89–103. 10.1016/j.bcp.2018.04.022 29684379

[B45] Márta K. Hasan P. Rodríguez-Prados M. Paillard M. Hajnóczky G. (2020). Pharmacological inhibition of the mitochondrial Ca2+ uniporter: relevance for pathophysiology and human therapy. J. Mol. Cell. Cardiol. 10.1016/j.yjmcc.2020.09.014. [Epub ahead of print]. 33035551 PMC7880870

[B46] Momose M. Iguchi N. Imamura K. Usui H. Ueda T. Miyamoto K.. (2001). Depressed myocardial fatty acid metabolism in patients with muscular dystrophy. Neuromuscul. Disord. 11, 464–469. 10.1016/S0960-8966(01)00191-2 11404118

[B47] Moore T. M. Lin A. J. Strumwasser A. R. Cory K. Whitney K. Ho T. (2020). Mitochondrial dysfunction is an early consequence of partial or complete dystrophin loss in mdx mice. Front. Physiol. 11:690 10.3389/fphys.2020.00690 32636760 PMC7317021

[B48] Naruse H. Miyagi J. Arii T. Ohyanagi M. Iwasaki T. Jinnai K. (2004). The relationship between clinical stage, prognosis, and myocardial damage in patients with Duchenne-type muscular dystrophy: five-year follow-up study. Ann. Nucl. Med. 18, 203–208. 10.1007/BF02985001 15233281

[B49] Nemani N. Dong Z. Daw C. C. Madaris T. R. Ramachandran K. Enslow B. T.. (2020). Mitochondrial pyruvate and fatty acid flux modulate MICU1-dependent control of MCU activity. Sci. Signal. 13:eaaz6206. 10.1126/scisignal.aaz6206 32317369 PMC7667998

[B50] Paillard M. Tubbs E. Thiebaut P.-A. Gomez L. Fauconnier J. Da Silva C. C.. (2013). Depressing mitochondria-reticulum interactions protects cardiomyocytes from lethal hypoxia-reoxygenation injury. Circulation 128, 1555–1565. 10.1161/CIRCULATIONAHA.113.001225 23983249

[B51] Pauly M. Angebault-Prouteau C. Dridi H. Notarnicola C. Scheuermann V. Lacampagne A.. (2017). ER stress disturbs SR/ER-mitochondria Ca2+ transfer: implications in Duchenne muscular dystrophy. Biochim. Biophys. Acta Mol. Basis Dis. 1863, 2229–2239. 10.1016/j.bbadis.2017.06.009 28625916

[B52] Pauly M. Daussin F. Burelle Y. Li T. Godin R. Fauconnier J.. (2012). AMPK activation stimulates autophagy and ameliorates muscular dystrophy in the mdx mouse diaphragm. Am. J. Pathol. 181, 583–592. 10.1016/j.ajpath.2012.04.004 22683340

[B53] Payne R. Hoff H. Roskowski A. Foskett J. K. (2017). MICU2 restricts spatial crosstalk between InsP 3 R and MCU channels by regulating threshold and gain of micu1-mediated inhibition and activation of MCU. Cell Rep. 21, 3141–3154. 10.1016/j.celrep.2017.11.064 29241542 PMC5734103

[B54] Percival J. M. Siegel M. P. Knowels G. Marcinek D. J. (2013). Defects in mitochondrial localization and ATP synthesis in the mdx mouse model of Duchenne muscular dystrophy are not alleviated by PDE5 inhibition. Hum. Mol. Genet. 22, 153–167. 10.1093/hmg/dds415 23049075 PMC3522404

[B55] Perloff J. K. Henze E. Schelbert H. R. (1984). Alterations in regional myocardial metabolism, perfusion, and wall motion in Duchenne muscular dystrophy studied by radionuclide imaging. Circulation 69, 33–42. 10.1161/01.CIR.69.1.33 6605817

[B56] Prosser B. L. Ward C. W. Lederer W. J. (2011). X-ROS signaling: rapid mechano-chemo transduction in heart. Science 333, 1440–1445. 10.1126/science.1202768 21903813

[B57] Quinlan J. G. Hahn H. S. Wong B. L. Lorenz J. N. Wenisch A. S. Levin L. S. (2004). Evolution of the mdx mouse cardiomyopathy: physiological and morphological findings. Neuromuscul. Disord. 14, 491–496. 10.1016/j.nmd.2004.04.007 15336690

[B58] Quinlivan R. M. Lewis P. Marsden P. Dundas R. Robb S. A. Baker E.. (1996). Cardiac function, metabolism, and perfusion in Duchenne and Becker muscular dystrophy. Neuromuscul. Disord. 6, 237–246. 10.1016/0960-8966(96)00007-7 8887952

[B59] Reagan-Shaw S. Nihal M. Ahmad N. (2008). Dose translation from animal to human studies revisited. FASEB J. 22, 659–661. 10.1096/fj.07-9574LSF 17942826

[B60] Rieusset J. Fauconnier J. Paillard M. Belaidi E. Tubbs E. Chauvin M.-A.. (2016). Disruption of calcium transfer from ER to mitochondria links alterations of mitochondria-associated ER membrane integrity to hepatic insulin resistance. Diabetologia 59, 614–623. 10.1007/s00125-015-3829-8 26660890

[B61] Rossini M. Filadi R. (2020). Sarcoplasmic reticulum-mitochondria kissing in cardiomyocytes: Ca2+, ATP, and undisclosed secrets. Front. Cell Dev. Biol. 8:532. 10.3389/fcell.2020.00532 32671075 PMC7332691

[B62] Rybalka E. Timpani C. A. Cooke M. B. Williams A. D. Hayes A. (2014). Defects in mitochondrial ATP synthesis in dystrophin-deficient mdx skeletal muscles may be caused by complex I insufficiency. PLoS ONE 9:e115763. 10.1371/journal.pone.0115763 25541951 PMC4277356

[B63] Saddik M. Gamble J. Witters L. A. Lopaschuk G. D. (1993). Acetyl-CoA carboxylase regulation of fatty acid oxidation in the heart. J. Biol. Chem. 268, 25836–25845. 7902355

[B64] Sasaki K. Sakata K. Kachi E. Hirata S. Ishihara T. Ishikawa K. (1998). Sequential changes in cardiac structure and function in patients with Duchenne type muscular dystrophy: a two-dimensional echocardiographic study. Am. Heart J. 135, 937–944. 10.1016/S0002-8703(98)70057-2 9630096

[B65] Scarpulla R. C. (2011). Metabolic control of mitochondrial biogenesis through the PGC-1 family regulatory network. Biochim. Biophys. Acta 1813, 1269–1278. 10.1016/j.bbamcr.2010.09.019 20933024 PMC3035754

[B66] Segawa K. Sugawara N. Maruo K. Kimura K. Komaki H. Takahashi Y.. (2020). Left ventricular end-diastolic diameter and cardiac mortality in Duchenne muscular dystrophy. Neuropsychiatr. Dis. Treat. 16, 171–178. 10.2147/NDT.S235166 32021209 PMC6972578

[B67] Sperl W. Skladal D. Gnaiger E. Wyss M. Mayr U. Hager J.. (1997). High resolution respirometry of permeabilized skeletal muscle fibers in the diagnosis of neuromuscular disorders. Mol. Cell. Biochem. 174, 71–78. 10.1023/A:1006880529195 9309668

[B68] Sun W. Liu Q. Leng J. Zheng Y. Li J. (2015). The role of Pyruvate Dehydrogenase Complex in cardiovascular diseases. Life Sci. 121, 97–103. 10.1016/j.lfs.2014.11.030 25498896

[B69] Suntar I. Sureda A. Belwal T. Sanches Silva A. Vacca R. A. Tewari D.. (2020). Natural products, PGC-1α, and Duchenne muscular dystrophy. Acta Pharm. Sin. B 10, 734–745. 10.1016/j.apsb.2020.01.001 32528825 PMC7276681

[B70] Tarasova N. V. Vishnyakova P. A. Logashina Y. A. Elchaninov A. V. (2019). Mitochondrial calcium uniporter structure and function in different types of muscle tissues in health and disease. Int. J. Mol. Sci. 20:4823. 10.3390/ijms20194823 31569359 PMC6801532

[B71] Ullrich N. D. Fanchaouy M. Gusev K. Shirokova N. Niggli E. (2009). Hypersensitivity of excitation-contraction coupling in dystrophic cardiomyocytes. Am. J. Physiol. Heart Circ. Physiol. 297, H1992–H2003. 10.1152/ajpheart.00602.2009 19783774 PMC3774091

[B72] Vial G. Detaille D. Guigas B. (2019). Role of mitochondria in the mechanism(s) of action of metformin. Front. Endocrinol. 10:294. 10.3389/fendo.2019.00294 31133988 PMC6514102

[B73] Viola H. M. Davies S. M. K. Filipovska A. Hool L. C. (2013). L-type Ca(2+) channel contributes to alterations in mitochondrial calcium handling in the mdx ventricular myocyte. Am. J. Physiol. Heart Circ. Physiol. 304, H767–H775. 10.1152/ajpheart.00700.2012 23335798

[B74] Vitiello L. Tibaudo L. Pegoraro E. Bello L. Canton M. (2019). Teaching an old molecule new tricks: drug repositioning for Duchenne muscular dystrophy. Int. J. Mol. Sci. 20:6053. 10.3390/ijms20236053 31801292 PMC6929176

[B75] Wang Y. An H. Liu T. Qin C. Sesaki H. Guo S.. (2019). Metformin improves mitochondrial respiratory activity through activation of AMPK. Cell Rep. 29, 1511–1523. 10.1016/j.celrep.2019.09.070 31693892 PMC6866677

[B76] Williams I. A. Allen D. G. (2007a). Intracellular calcium handling in ventricular myocytes from mdx mice. Am. J. Physiol. Heart Circ. Physiol. 292, H846–H855. 10.1152/ajpheart.00688.2006 17012353

[B77] Williams I. A. Allen D. G. (2007b). The role of reactive oxygen species in the hearts of dystrophin-deficient mdx mice. Am. J. Physiol. Heart Circ. Physiol. 293, H1969–H1977. 10.1152/ajpheart.00489.2007 17573457

[B78] Wu S. Lu Q. Ding Y. Wu Y. Qiu Y. Wang P.. (2019). Hyperglycemia-driven inhibition of AMP-activated protein kinase α2 induces diabetic cardiomyopathy by promoting mitochondria-associated endoplasmic reticulum membranes *in vivo*. Circulation 139, 1913–1936. 10.1161/CIRCULATIONAHA.118.033552 30646747 PMC6465113

