# Supplementary figures and images for: Metformin Reverses the Enhanced Myocardial SR/ER–Mitochondria Interaction and Impaired Complex I-Driven Respiration in Dystrophin-Deficient Mice
Source: Front Cell Dev Biol. 2021 Jan 25;8:609493. doi: 10.3389/fcell.2020.609493 (PMC7868535; doi:10.3389/fcell.2020.609493)

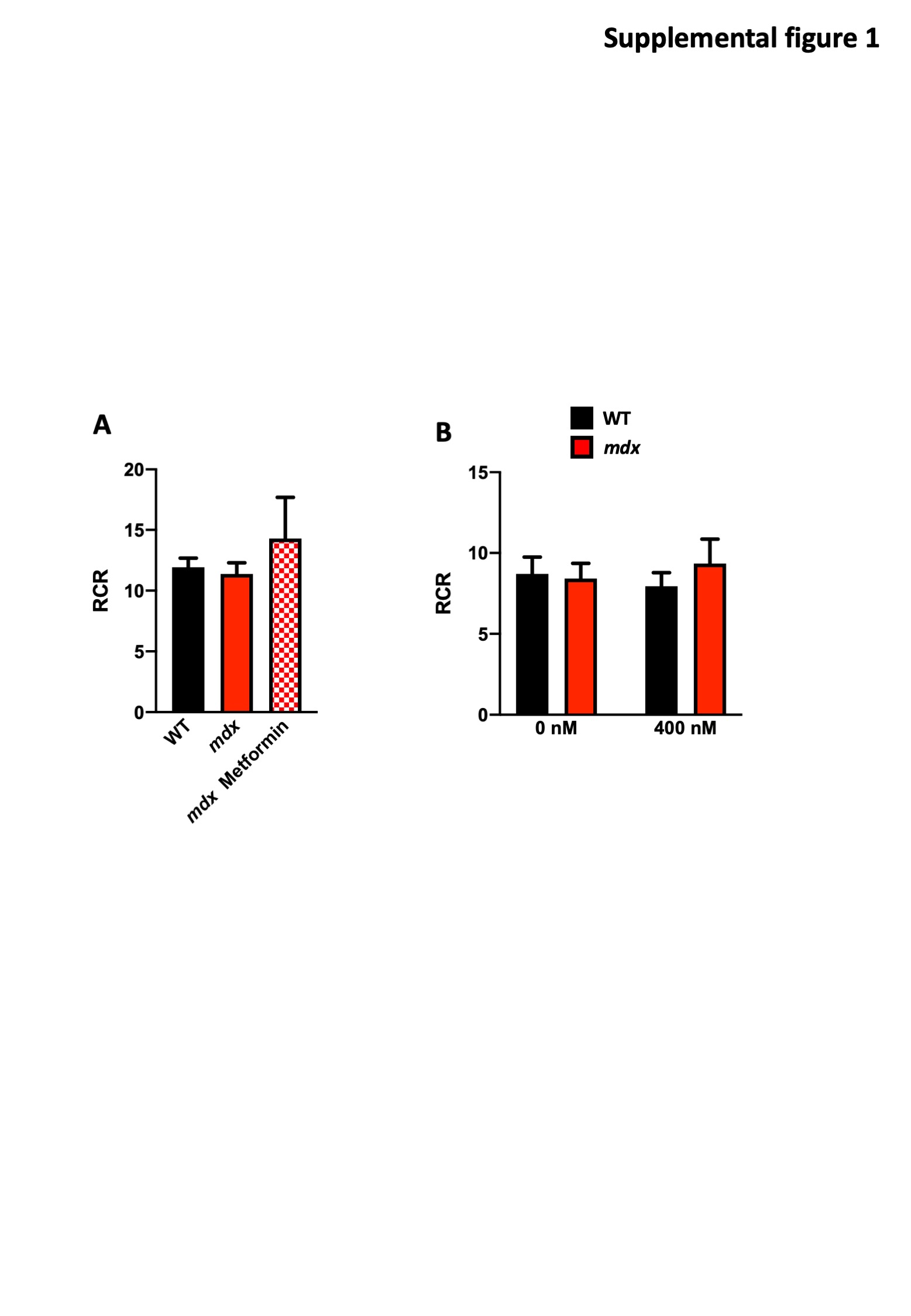

Supplement: Supplementary Figure 1 — Respiratory control ratio (RCR) was measured in (A) WT (N = 7), mdx (N = 13) and mdx + met (N = 9) hearts and (B) in WT (N = 6) and mdx (N = 6) in presence of in presence of 0 nM or 400 nM extramitochondrial Ca2+. Data are mean ± SEM, p > 0.05 mdx vs. WT. [file Image_1.JPEG]

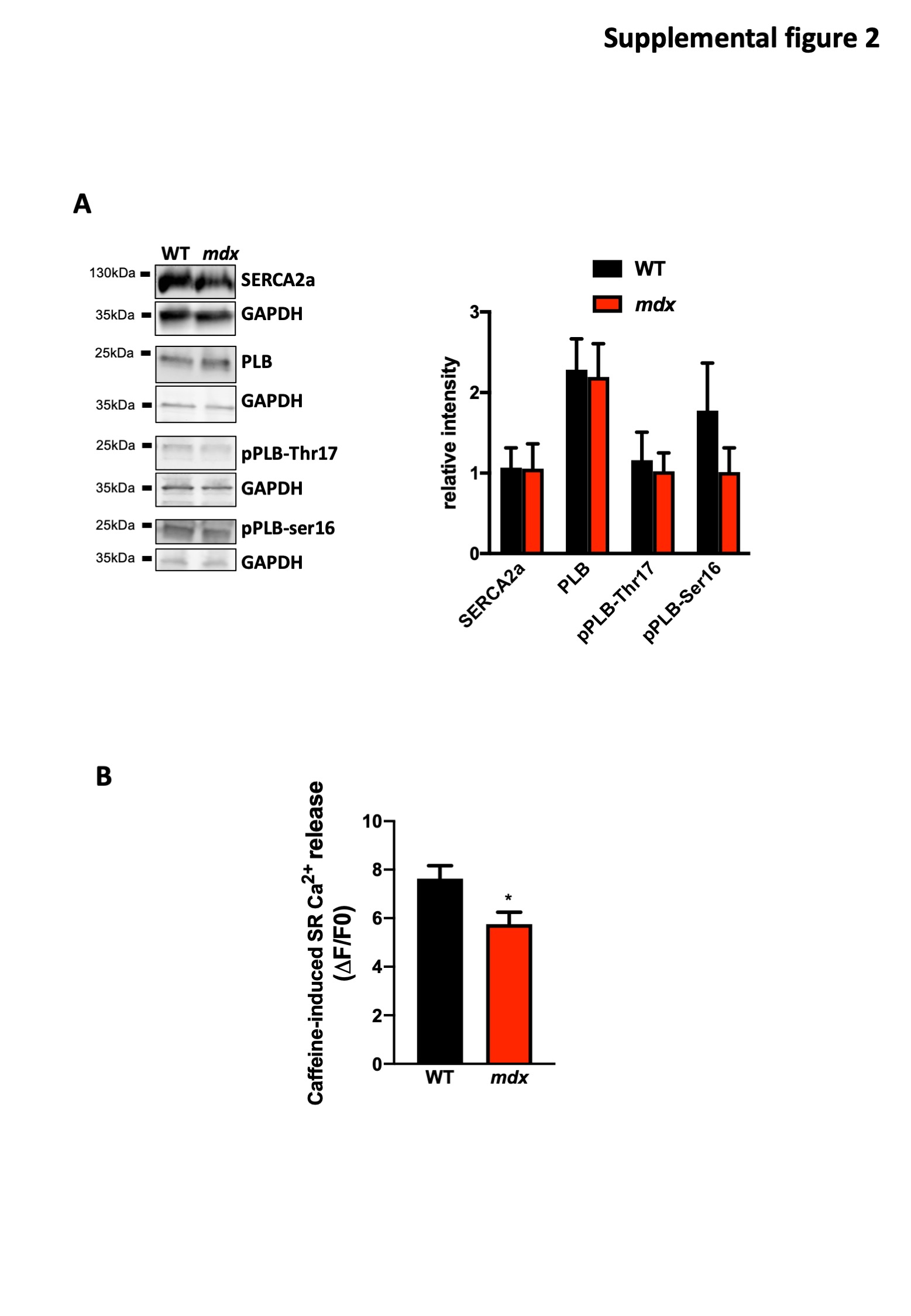

Supplement: Supplementary Figure 2 — SERCA2a, PLB expression, and SR Ca2+ load. (A) Full length immunoblots and quantification of SERCA2a, phospholamban (PLB), and Phospho-Phospholamban (Ser16/Thr17) (pPLB-Ser16; pPLB-Thr17) were normalized to GAPDH. Data are mean ± SEM, p > 0.05 mdx (N = 4-6) vs. WT (N = 4-6). (B) Mean values of the amplitude of caffeine-induced SR Ca2+ release, estimating the SR Ca2+ load. *p = 0.0182 WT (N = 3; N = 9) vs. mdx (N = 3; N = 10). [file Image_2.JPEG]

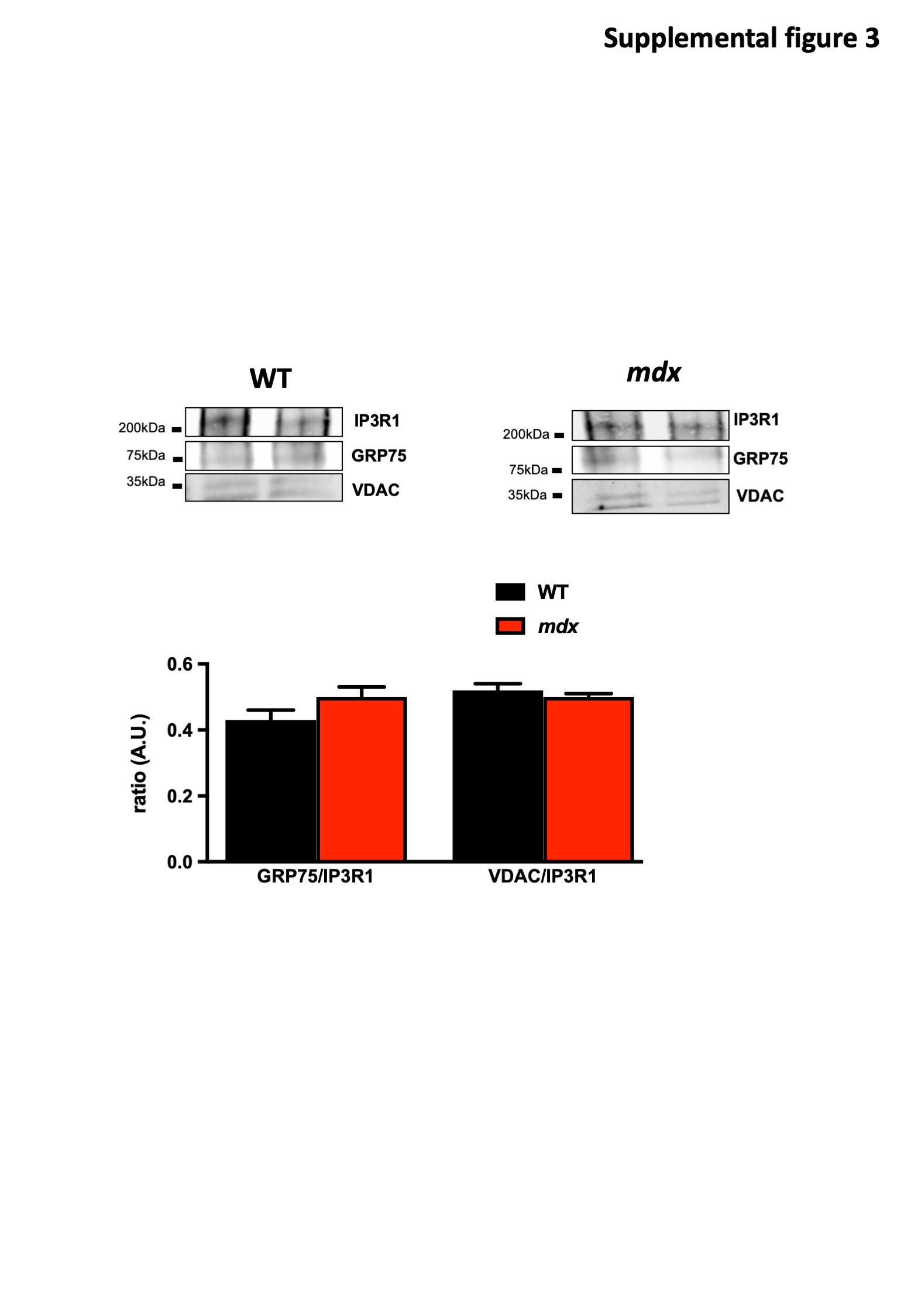

Supplement: Supplementary Figure 3 — IP3R1 immunoprecipitation. Anti-IP3R1 antibody (1:200) was used to immunoprecipitate IP3R1 from heart homogenate. Samples were incubated with an anti-IP3R1 antibody in 0.5 ml of a modified RIPA buffer (10 mM Tris–HCl, pH 7.4; 150 mM NaCl; 1% Triton; 5 mM NaF and protease inhibitor cocktail) for 2 h at 4°C. The immune complex was incubated with protein A/G magnetic beads (Pierce 88802) at 4°C for 2 h, after which the beads were washed out three times with RIPA buffer. Proteins were separated on SDS/PAGE gels and transferred onto nitrocellulose membranes for 1 h at 100 V. The immunoblots were prepared using antibodies against IP3R1 (1:1,000), anti-GRP75 antibody (1:1,000, Santa Cruz) and anti-VDAC (1:300). All immunoblots were developed and quantified using the Odyssey infrared imaging system (LICOR Biosystems) and infrared-labeled secondary antibodies. After immunoprecipitation (IP) of IP3R1 from heart muscle of WT or mdx mice, immunoblots were used to detect IP3R1, Grp75, and VDAC. Data are mean ± SEM, p > 0.05 mdx (N = 3) vs. WT (N = 3). [file Image_3.JPEG]

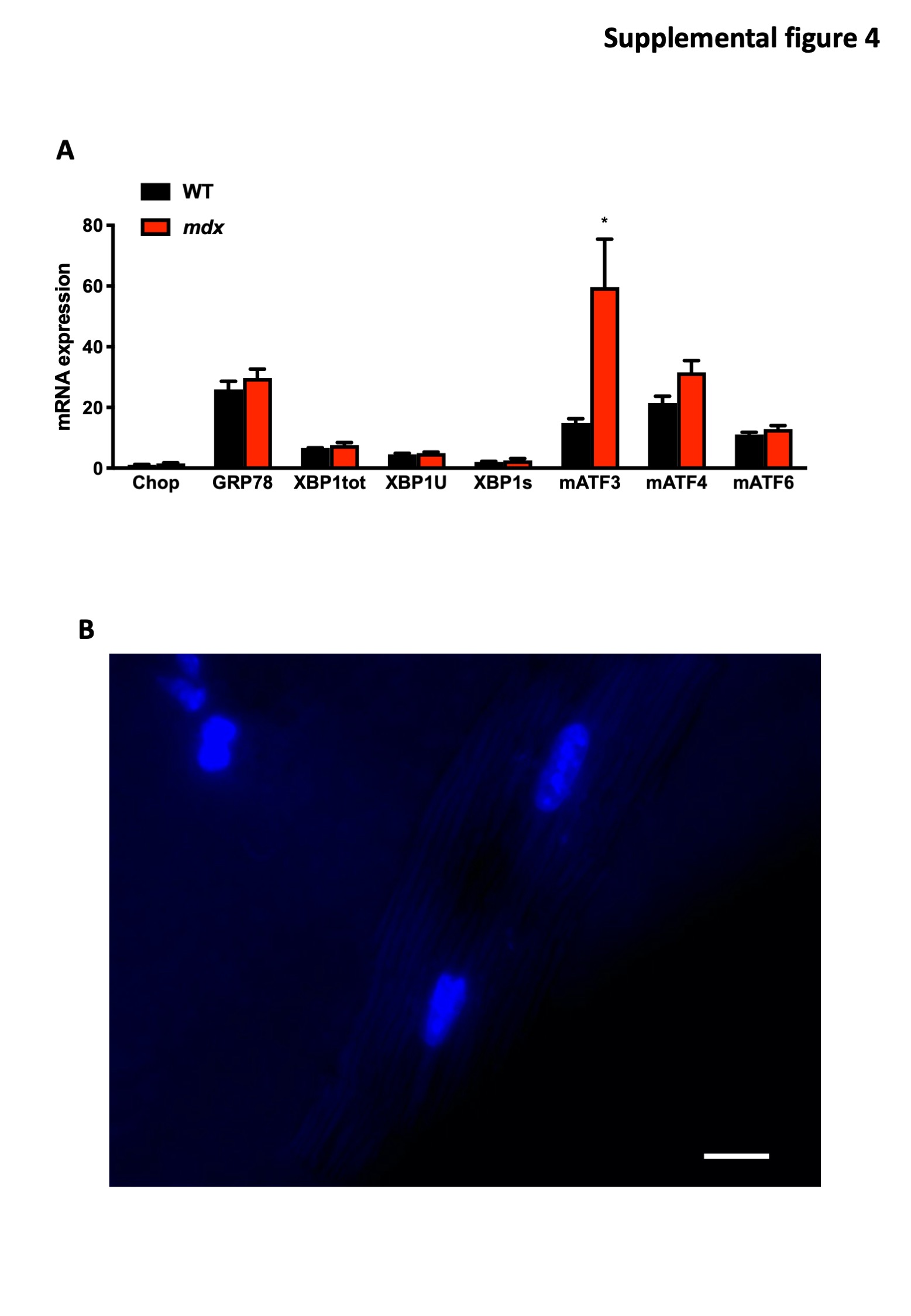

Supplement: Supplementary Figure 4 — (A) Transcript levels of UPR response genes quantified by 30 reverse transcription quantitative polymerase chain reaction (RT-qPCR) in WT (N = 4) and mdx (N = 4) hearts. The mRNA levels were normalized to the reference gene TBP. Data are means ± SEM, *p = 0.0286 mdx vs. WT. (B) PLA technical negative control obtained in absence of primary antibody. [file Image_4.JPEG]

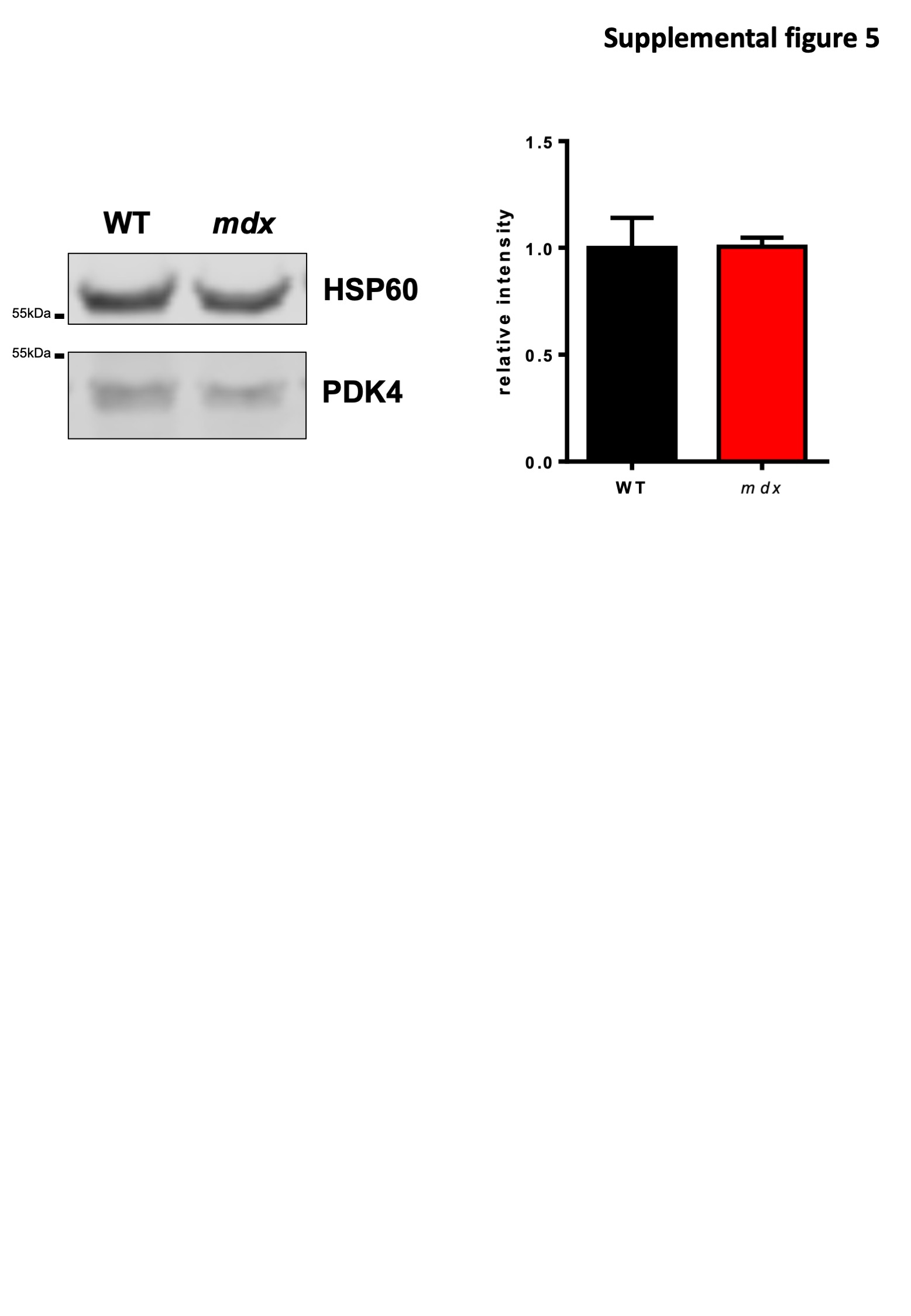

Supplement: Supplementary Figure 5 — Pyruvate dehydrogenase kinase 4 (PDK4) expression. Full length immunoblots and quantification of PDK4 were normalized to Hsp60. Data are mean ± SEM, p > 0.05 mdx (N = 5) vs. WT (N = 5). [file Image_5.JPEG]

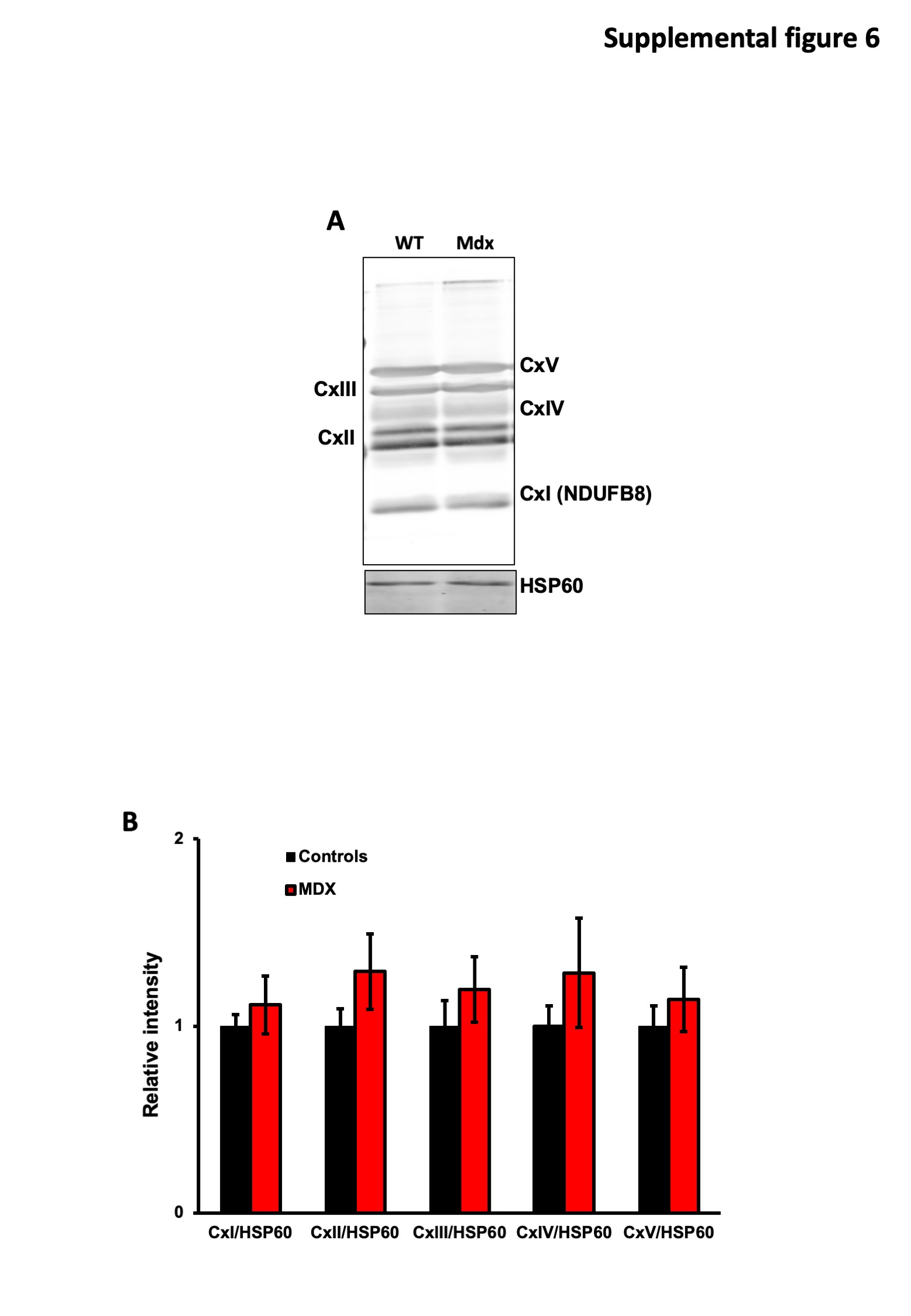

Supplement: Supplementary Figure 6 — Protein expression of mitochondrial respiratory chain complexes. All western blots were performed on isolated mitochondria and quantifications of proteins are normalized to Hsp60. (A) Representative immunoblots and (B) quantification of complex I (CxI), complex 40 II(CxII), complex III (CxIII), complex IV (CxIV), complex V (Cx V). Data are mean ± SEM, mdx (N = 6) vs. WT (N = 6). [file Image_6.JPEG]

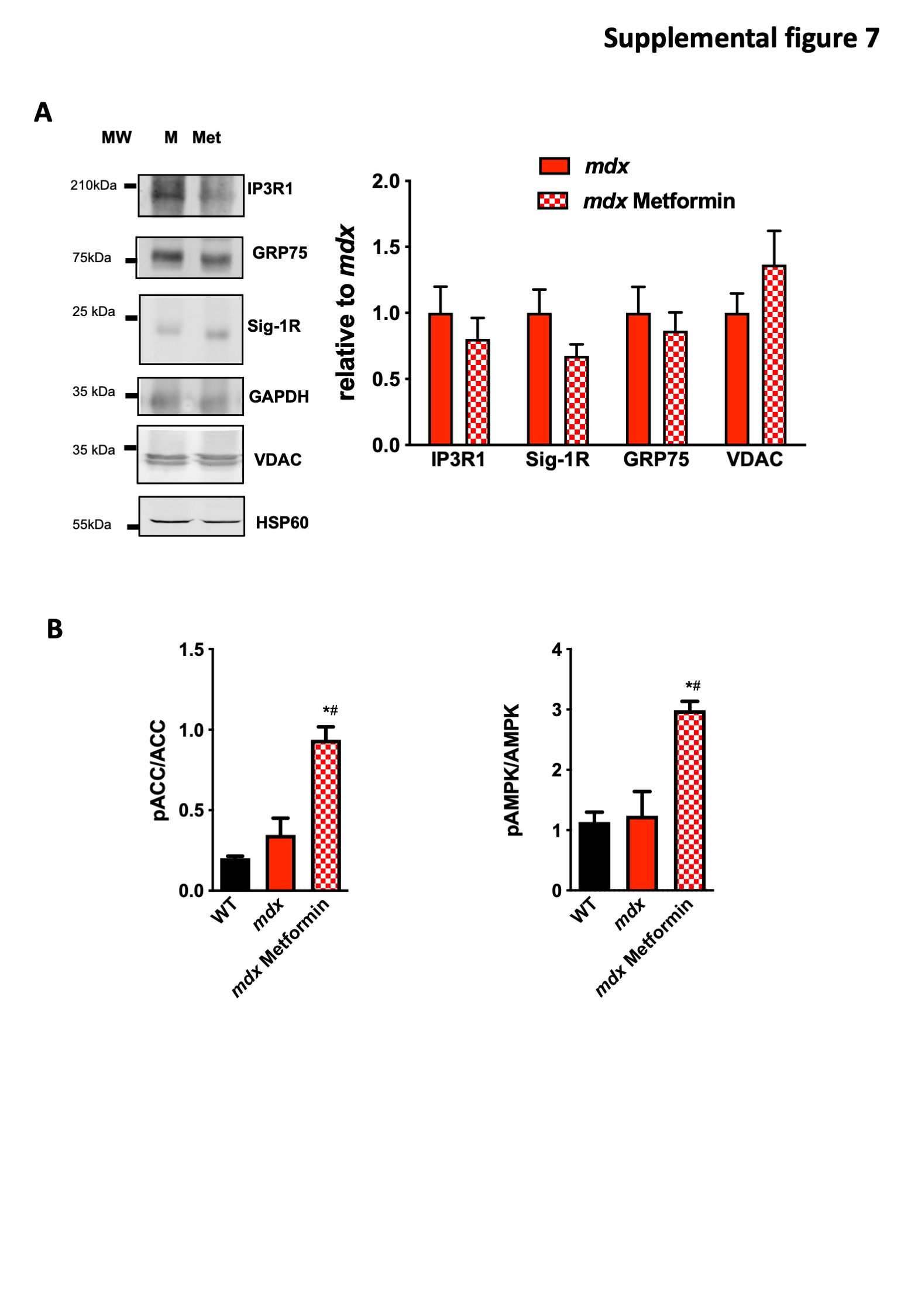

Supplement: Supplementary Figure 7 — Phosphorylation level of AMPK and ACC, and proteins expression of SR/ER and mitochondria contacts points. All quantifications of proteins are normalized either to GAPDH or Actin when heart homogenates were used or to Hsp60 when western blot were performed on isolated mitochondria. (A) Representative immunoblots and quantification of IP3R1, GRP75, Sig-1R, and VDAC. Quantification of IP3R1, GRP75, MFN2, and Sig-1R was carried out from N = 6 mdx and mdx +metformin hearts. Data are mean ± SEM. (B) Mean value of pAMPK/AMPK and pACC/ACC ratio. Ratio were established after proteins normalization to Actin from N = 6 WT, mdx and mdx +metformin hearts. Full lengths gels are in supplemental information. WT vs. mdx+ metformin #p = 0.0101 and mdx vs. mdx+metformin *p = 0.0419. [file Image_7.JPEG]

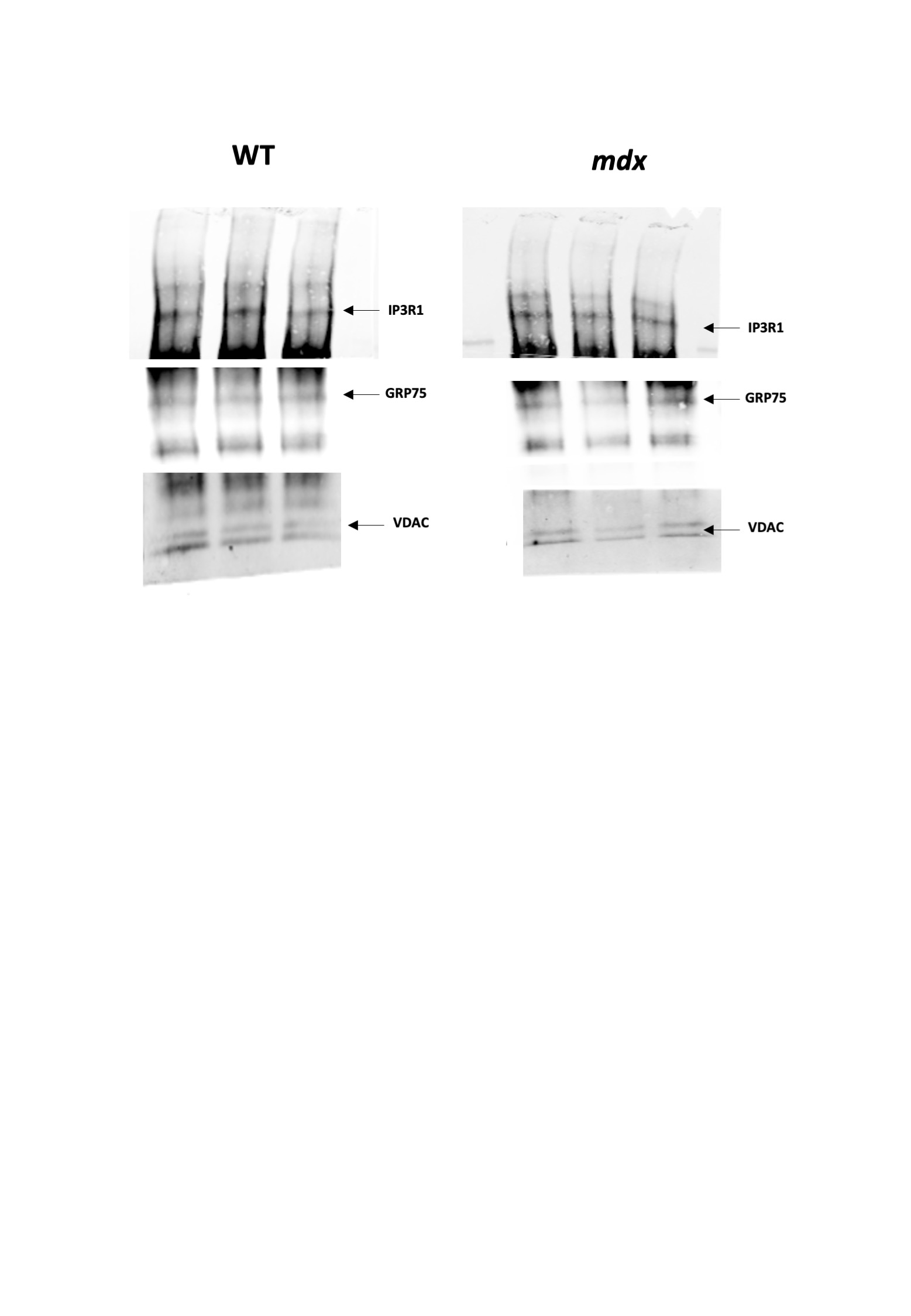

Supplement: Supplementary Information — All full lengths gel for all protein probed. [file Image_8.JPEG]
